# A versatile toolbox for semi-automatic cell-by-cell object-based colocalization analysis

**DOI:** 10.1038/s41598-020-75835-7

**Published:** 2020-11-04

**Authors:** Anders Lunde, Joel C. Glover

**Affiliations:** Laboratory of Neural Development and Optical Recording (NDEVOR), Division of Physiology, Department of Molecular Medicine, University of Oslo, Blindern, 1105, Oslo, Norway

**Keywords:** Image processing, Molecular biology

## Abstract

Differential fluorescence labeling and multi-fluorescence imaging followed by colocalization analysis is commonly used to investigate cellular heterogeneity in situ. This is particularly important when investigating the biology of tissues with diverse cell types. Object-based colocalization analysis (OBCA) tools can employ automatic approaches, which are sensitive to errors in cell segmentation, or manual approaches, which can be impractical and tedious. Here, we present a novel set of tools for OBCA using a semi-automatic approach, consisting of two ImageJ plugins, a Microsoft Excel macro, and a MATLAB script. One ImageJ plugin enables customizable processing of multichannel 3D images for enhanced visualization of features relevant to OBCA, and another enables semi-automatic colocalization quantification. The Excel macro and the MATLAB script enable data organization and 3D visualization of object data across image series. The tools are well suited for experiments involving complex and large image data sets, and can be used in combination or as individual components, allowing flexible, efficient and accurate OBCA. Here we demonstrate their utility in immunohistochemical analyses of the developing central nervous system, which is characterized by complexity in the number and distribution of cell types, and by high cell packing densities, which can both create challenging situations for OBCA.

## Introduction

The central nervous system (CNS) is characterized by the exceptional molecular, morphological and physiological diversity of its constituent neurons^1,2^. Much progress has been made in relating distinct patterns of gene and protein expression to neuronal structure and function. However, the full molecular diversity of neuron types is far from completely described, even in intensively studied model organisms.

Multi-fluorescence imaging is used extensively to study the correlation of molecular, structural and functional features in neurons^3–7^. Multi-fluorescence imaging involves the differential fluorescent labeling of neurons using a variety of techniques, including retrograde axonal tracing, immunolabeling, transgene-driven expression of fluorescent reporter proteins, and fluorescently tagged endogenous proteins^8–10^. RNA expression can also be visualized by fluorescence in-situ hybridization^11^. Tissue samples can be processed with combinations of these techniques and subsequently imaged, allowing researchers to directly relate the different labeled components. With the exception of axonal tracing, the same techniques are also used to study molecular diversity in many non-neuronal tissues. Advances in multi-fluorescence imaging, including automated whole section microscopy and three-dimensional imaging, have generated increasingly more complex data sets. Accordingly, analyzing the data can represent a major bottleneck in the research pipeline.

To analyze multi-fluorescence imaging data, researchers commonly employ colocalization analysis, which seeks to quantify the degree to which different fluorescent labels overlap. Colocalization analysis usually necessitates an initial segmentation of the image into biologically meaningful regions of interest, e.g. cells, nuclei, organelles, or synapses. Following segmentation, several different automatic colocalization analysis algorithms can be applied for quantification. These can be categorized as either pixel-based or object-based (reviewed in^4,12,13^).

Pixel-based algorithms are used to calculate correlation or overlap coefficients within delineated regions of interest, by comparing pixel-to-pixel brightness values in two or more imaging channels. This approach is often used to study molecular interactions inside of cells, since colocalization features of individual pixels are quantified. The benefits of pixel-based analysis include ease of use and rigorously defined coefficients that enable objective quantification of signal colocalization at the pixel level. However, image noise and artifacts can skew or perturb coefficients, although methods to counteract the influence of image noise exist. A related method, although not normally classified as pixel-based, performs colocalization analysis by calculating aggregate pixel value metrics (e.g. mean brightness) inside regions of interests (e.g. cells) across image channels. Such metrics can be used to define the presence or absence of fluorescent labeling for each region, or even the level of labeling.

In contrast, object-based colocalization analysis (OBCA) algorithms depend on locating and defining objects of interest across image channels, typically as fully delineated objects or as centroids. Colocalization can subsequently be quantified using a variety of metrics, for example the degree of pixel overlap between delineated objects, or distances between the centroids of neighboring objects. OBCA is less sensitive to image noise, although image noise can influence successful localization of objects. A notable application of OBCA that is not satisfied by pixel-based algorithms is determining which cells co-express markers that do not overlap in space, for example nuclear versus cytoplasmic markers.

A critical step to ensure the success of OBCA is accurate image segmentation. To automate segmentation, a much used and simple first step is based on intensity thresholding, in which image pixels are classified as being either part of the relevant object set or part of the image background, based on pixel brightness values^14,15^. In the resulting binary image, the object set can be further segmented into individual objects by grouping together connected pixels, which become the basis for object delineations. For centroid based approaches, centroids can be calculated from delineations, but can also be estimated directly from grayscale images by algorithms that detect local maxima of pixel intensity. Several more advanced segmentation algorithms have been described that improve performance in cases where separation of objects is difficult (reviewed in^15,16^).

Tools and methods that employ fully automatic colocalization analysis have the benefit of providing reproducibility and speed, albeit sometimes at the cost of accuracy. A major reason for lowered accuracy is that full automation of segmentation and object identification is a notoriously difficult problem to solve, especially for complex objects such as neurons^15,16^. To address the need for a high-throughput OBCA workflow that does not rely exclusively on fallible automated algorithms and that leverages human visual processing capacity, we have developed a set of tools for semi-automatic OBCA that combines automation for speed with visual/manual verification for accuracy. The tools we present use image binarization and other operations to extract and visualize meaningful colocalization signals, but ultimate quantification is based on a centroid-like approach in which objects are defined by a single point. We emphasize the utility of visual verification and correction of automatic centroid placement, without the need to perform time consuming corrections to object delineations for quantification.

**Figure 1:**
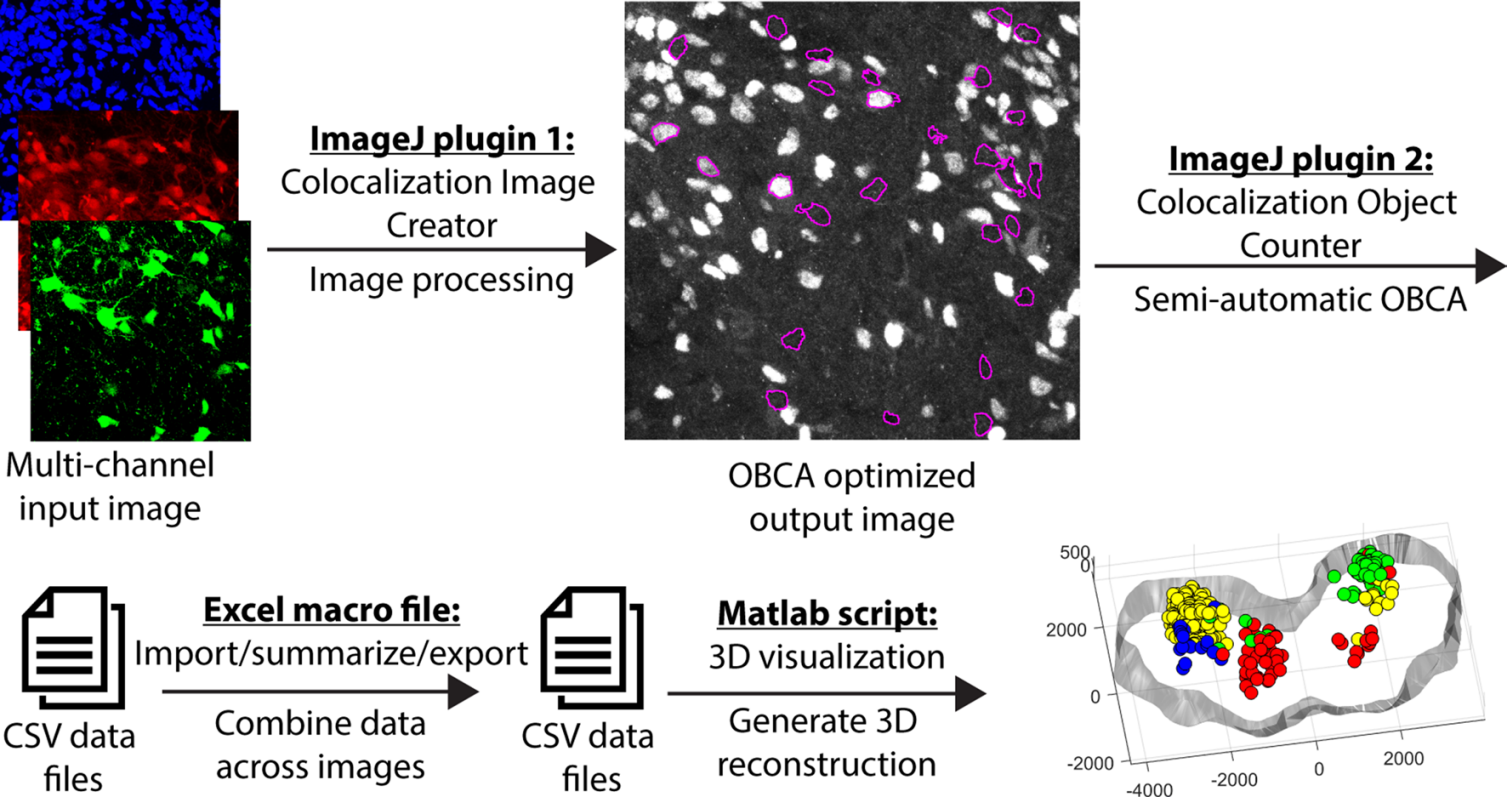
Schematic of OBCA workflow. Each plugin/script in the workflow generates output data that can be further analyzed by one of the other plugins/scripts in the flow. The ImageJ Colocalization Image Creator generates new images enhanced for semi-automatic OBCA. The ImageJ Colocalization Object Counter enables semi-automatic OBCA, and generates data files with counts and colocalization information in comma separated value (csv) format. The Excel macro summarizes, organizes and exports the data in combined csv data files. The MATLAB script reads the data files and generates an interactive 3D reconstruction. All four plugins/scripts can function as stand-alone tools, or combined as desired.

**Figure 2:**
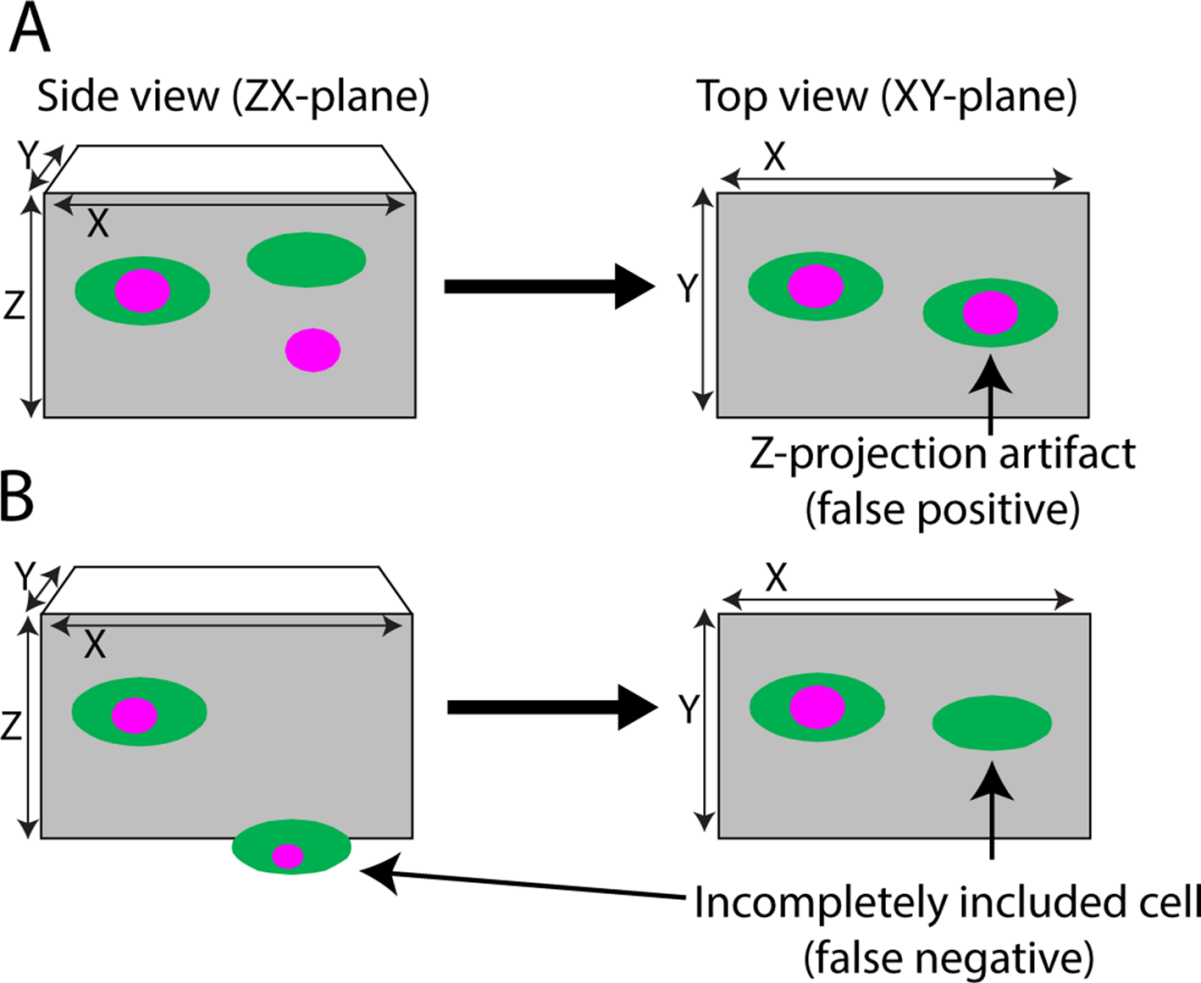
Z-projection artifacts and incompletely included cells can lead to false positive and false negative colocalization. Cartoons of tissue sections, on the left viewed from the side, and on the right viewed from the top, as indicated by the X, Y and Z axes. Green ovals represent cell bodies with a cytoplasmic label, and magenta circles represent a labeled subcellular structure, e.g. a cell nucleus. (**A**) There is no colocalization of the two labels on the right when viewed in the ZX plane, but in the commonly used XY-plane a Z-projection artifact arises, presenting as a double-labeled cell. (**B**) The cell to the right is only partly included, with only the green cytoplasmic signal present in the section. Here, a false negative would arise irrespective of the angle of view. False observations can also occur when both labels are distributed throughout the cell if one signal is weak and goes undetected in cells only partially contained within the section.

A schematic of the tool-chain workflow is shown in Fig. 1. The entry point to the workflow consists of a plugin (the Colocalization Image Creator) for the popular free and open source image analysis software ImageJ^17^. The Colocalization Image Creator enables flexible processing of image data into a visual format that is better suited to high-throughput semi-automatic OBCA. It can produce processed binary and grayscale signal outputs, visualize signal overlap across channels, and generate a special Z-projection where 3D information is condensed onto a 2D plane for easy visualization of 3D colocalization data, in a way that minimizes Z-projection artifacts (examples of such artifacts are shown in Fig. 2A). Additionally, signal overlap processing enables restricting visualization to labeled cellular sub-compartments, for example cell nuclei, which can improve object segmentation. This can also minimize artifacts that arise from partially transected objects (example in Fig. 2B).

A second ImageJ plugin (the Colocalization Object Counter) enables OBCA quantification in any type of image. It uses local maxima algorithms to automatically define objects as single points, which can be edited and verified visually in a semi-automatic manner. The Colocalization Object Counter enables annotating which label(s) are associated with each object (object *colocalization category*). The plugin also contains basic tools for subsequent 3D reconstruction of object and tissue contour data.

A third tool, in the form of a Microsoft Excel macro file, enables data import overview, revision, statistical analysis, and export. Exported data can be imported by a fourth tool, a Matlab script that enables interactive 3D visualization of identified objects with their colocalization categories indicated within visible tissue contours.

Although we have designed and optimized this tool set for semi-automatic OBCA of multi-fluorescence imaging data from the developing central nervous system, the workflow is adaptable to other types of imaging data as well. The tools complement each other, but can also be used individually. Below, we describe the function of each tool, provide some examples of usage, and provide an assessment of robustness across individual operators. We compare our platform head-to-head with similar platforms, and we evaluate specific limitations associated with our platform. In the “Supplementary Information” we provide specific details on the operation and user interfaces of the tool set.

## Results

### ImageJ plugin 1: Colocalization Image Creator

#### Purpose, input requirements, and output

The colocalization image creator plugin allows the creation of custom binary or grayscale visualizations based on raw data of input multichannel images, into a format that is more suitable for semi-automatic OBCA. The output visualizations can be based on a single input channel, or represent signal overlap across input channels, thus providing the opportunity to assess which markers any labeled cell or cell compartment contains. The visualizations and the markers they represent are assigned in the form of *image elements* (see below), which are created during plugin operation. An important feature is that the flexibility of the plugin facilitates visualization of cellular marker colocalization in various ways, accommodating different experimental frameworks.

The input to the plugin must be a multichannel image in a format that is readable by ImageJ. Both 3D (Z-stacks) and 2D (single plane) images are accepted. When a single plane image is the input, the output is a processed single plane image. When Z-stacks are the input, the output is a processed Z-stack plus an additional special Z-projection that condenses 3D colocalization data into a single plane for ease of visualization. This special Z-projection is principally useful for visualizing 3D image data in a Z-stack with a limited Z-axis depth, since the image complexity increases as depth increases. Appropriately sized Z-stacks can be produced directly during image acquisition of sections, or be obtained from thicker 3D image data, for example from cleared tissue blocks, by digitally extracting optical sections at desired z-positions. The special Z-projection is inserted as a single plane image on top of the output Z-stack. The purpose of the special Z-projection is to simplify colocalization analysis and reduce the time spent, by permitting quick inspection during semi-automatic OBCA quantification, with inspection of the underlying Z-stack being performed only if necessary or desired (see plugin 2 for the quantification process). The special Z-projection is designed to algorithmically remove Z-projection artifacts (Fig. 2A). It is important to realize, however, that effective removal of Z-projection artifacts requires that the Z-stack input is generated with sufficient resolution, in particular in the Z-axis (optical slice thickness). See the “Supplementary Information” section online entitled “ImageJ plugin 1” for specific details on the plugin operation and user interfaces.

#### Image elements

At plugin startup, the output image is initially empty of contents. To start building an output image the user can add one or more *image elements* to the output by clicking the “Add element” button in the plugin main menu (Supplementary Fig. S1 online), which opens the *add image element wizard*. The image element is a central concept in the plugin, and is generated from a single or several input channels. The input channels are processed in various user-specified ways, for example restricting output to show overlapping signals across channels, applying ImageJ image filters, and setting the image element pixel color. By adding one or more image elements to the output, various colocalization relationships between input channels can be visualized.

**Figure 3:**
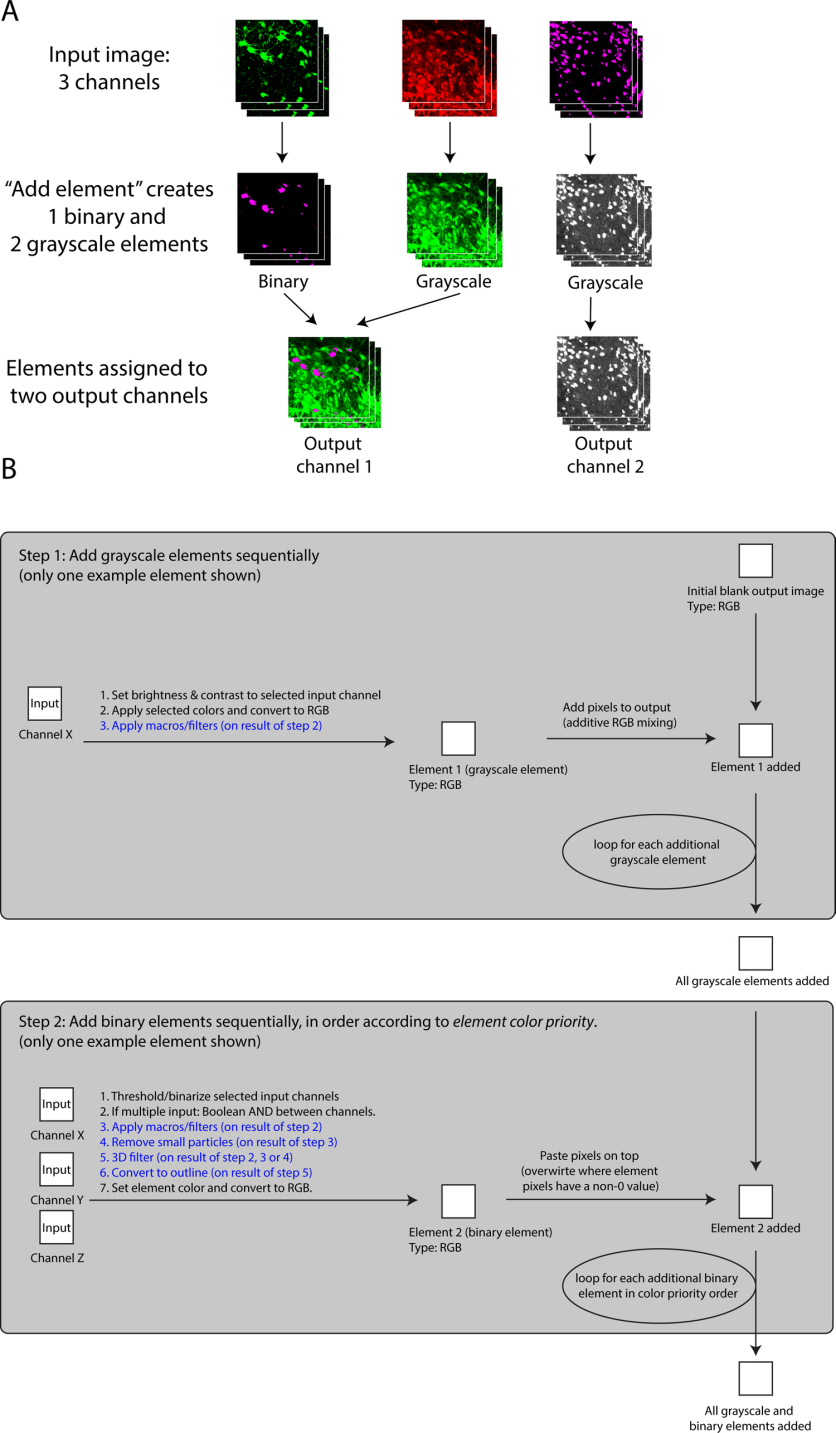
Example and Flowchart of Colocalization Image Creator processing algorithm. (**A**) Illustration of an example of Colocalization Image Creator output. In this example, the input image has three channels of Z-stacks, shown in the top row. From the input channels, three image elements are created, generated by using the “add element” wizard (Supplementary Fig. S1 online) three times. This creates one binary and two grayscale elements, shown in the middle row. Two of the image elements are assigned to output channel 1 in the wizard, whereas one of the elements is assigned to output channel 2, shown in the bottom row. The final output image in this example will thus be an image with two output channels. (**B**) Flowchart showing the main steps of the image element processing algorithm of the Colocalization Image Creator. Since every output channel is processed separately, the schematic shows how elements assigned to the same output channel are processed. The top gray square details step 1, where grayscale elements are added to the output image. The bottom gray square details step 2, where binary elements are added, thus overwriting any signals from the grayscale elements. Note that binary elements are added in order according to the element’s color priority, ensuring that colors with the highest priority are added last, and thereby prioritized in the output (due to overwriting). Steps described with a blue font represent optional processing steps.

The output image has one channel initially, and image elements are by default assigned to that channel. There, colocalization can be evaluated from image element signals representing signal overlap. It is also possible to assign image elements to new output channels, thus creating multi-channel output images (Fig. 3A). Colocalization can thus alternatively or additionally be evaluated by comparing signals across output channels and assessing whether pairs of objects have the same XYZ locations across channels. This is useful for example if the user creates many image elements that could clutter visualization in a single output channel, or that could overwrite each other and thus obscure information or (2) creates “verification channels” (see the section entitled “Verification channels”).

There are two types of image elements, grayscale and binary, which differ in how and in what order they are processed and added to the output image by the element processing algorithm (Fig. 3B). Rules that define how multiple image elements interact with each other (for example whether element pixels blend colors, or overwrite each other) are enforced by the plugin through the element processing algorithm (schematically represented in Fig. 3B, and further described in the sections entitled “Element mixing rules” and “The Z-projection output”). These rules are designed to avoid common visual errors and colocalization artifacts, and are different for grayscale and binary elements.

#### Element type 1: Grayscale elements

**Figure 4:**
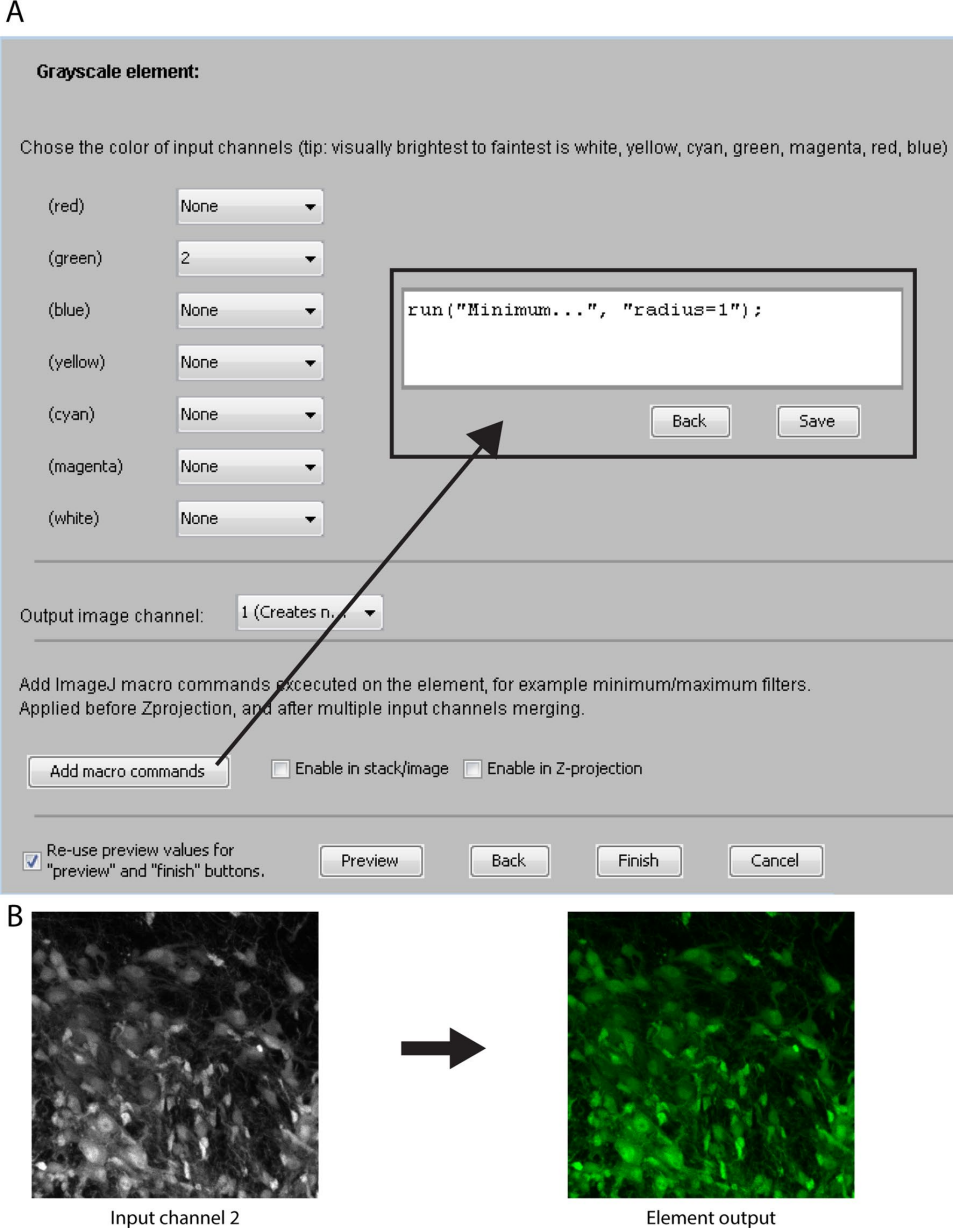
Menu for creating grayscale elements and example of output. (**A**) The menu for creating grayscale elements allows the user to select a specific color for each input image channel. The output channel is assigned with the drop down menu labeled “Output image channel”. Macro commands are added with the “Add macro commands” button. Clicking “Preview” displays a temporary preview of the grayscale element using the selected settings. (**B**) An example of a grayscale element output obtained by applying the settings shown in (**A**).

Grayscale element input channels are brightness adjusted, pseudo-colored, and optionally filtered or macro-transformed. The order of operations is shown in Fig. 3B (top section). One or more input channels can be selected to become part of the element output. In the case of using multiple input channels, additive mixing of the pixel values between input channels is performed. However, due to problems associated with Z-projection artifacts (Fig. 2A) and faint grayscale signals^12^, mixing multiple grayscale input channels is highly discouraged in the plugin, and will result in a pop-up warning. Grayscale elements are instead primarily useful for visualizing the signal of single input channels in cases where (a) binary segmentation is unreliable, (b) the intensity of the signal itself is of interest, or (c) when creating “verification channels”. However, single grayscale elements can effectively be combined with one or multiple *binary elements*, especially with binary elements that have the *outline* option enabled (see “Element type 2: binary elements” below), allowing visualization of signal overlap between binary and grayscale elements. Figure 4B shows an example of an input image channel being transformed to a grayscale element by applying the settings shown in Fig. 4A. See the “Supplementary Information” section online entitled “Element type 1: Grayscale elements” for details on the user interface for creating grayscale elements.

#### Element type 2: binary elements

**Figure 5:**
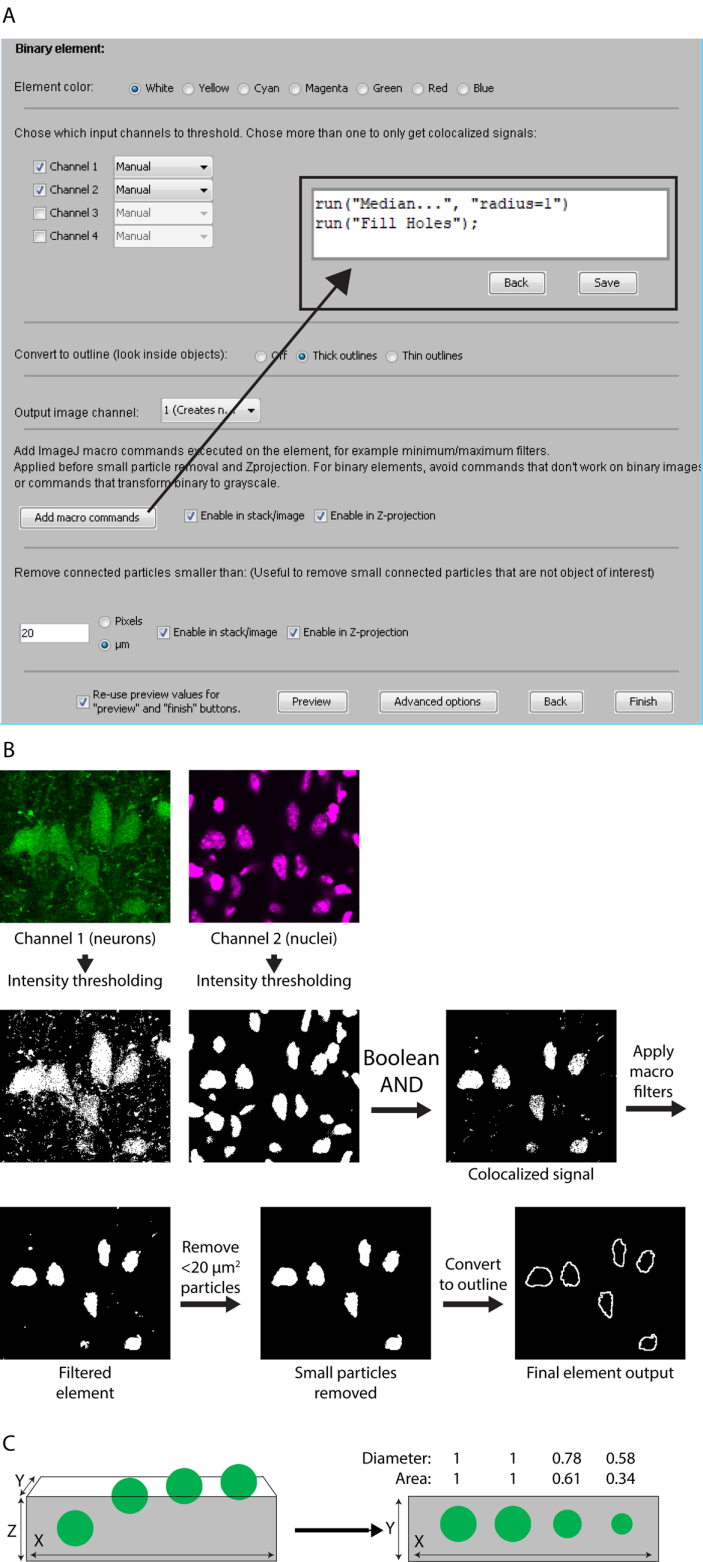
Menu for creating binary elements and example of output. (**A**) The menu for creating binary elements allows the user to select multiple input image channels, which are manually or automatically binarized and treated with the Boolean AND operation. The element color is selected near the top. An output image channel is assigned in a drop down menu. Macro commands are added using the “Add macro commands” button. Small particles in the image below a specified area are filtered away by defining a minimum area size. The “Advanced options” menu enables the user to select input channels that are subtracted from the element output following binarization. Clicking “Preview” displays a temporary preview of the binary element using the selected settings. (**B**) shows the sequence of processing steps and the final element output using the settings shown in (**A**). (**C**) Illustration of a set of spherical objects that are increasingly less included in a tissue section, thus generating profiles of increasingly smaller area. On the left, the four green circles represent objects with different degrees of inclusion in the tissue section, seen at the XZ-plane. On the right, the same four objects are shown as they would appear in the XY-plane, with their diameters and areas indicated relative to a fully included object of the same size.

Binary element input channels are thresholded/binarized, Boolean AND combined, optionally filtered/macro-transformed, optionally processed for small particle removal, optionally 3D object-filtered, optionally converted to outline, and pseudo-colored. The order of operations is shown in Fig. 3B (bottom section), the element creation user interface is shown in Fig. 5A, and an example output is shown in Fig. 5B. The plugin only offers global automatic or manual intensity thresholding for binarization. More advanced segmentation algorithms, if desired, would have to be pre-applied to input channels. When more than one input channel is selected, the Boolean AND operation is performed between the binarized channels, resulting in an output that only shows the overlapping pixels. For example, when imaging cell nuclei and different populations of labeled cells in separate input channels, binary elements can be used to visualize the nuclear area of specific cell types, and/or the region of overlap of specific cell types. See the “Supplementary Information” section online entitled “Element type 2: Binary elements” for details on the user interface for creating binary elements.

**Figure 6:**
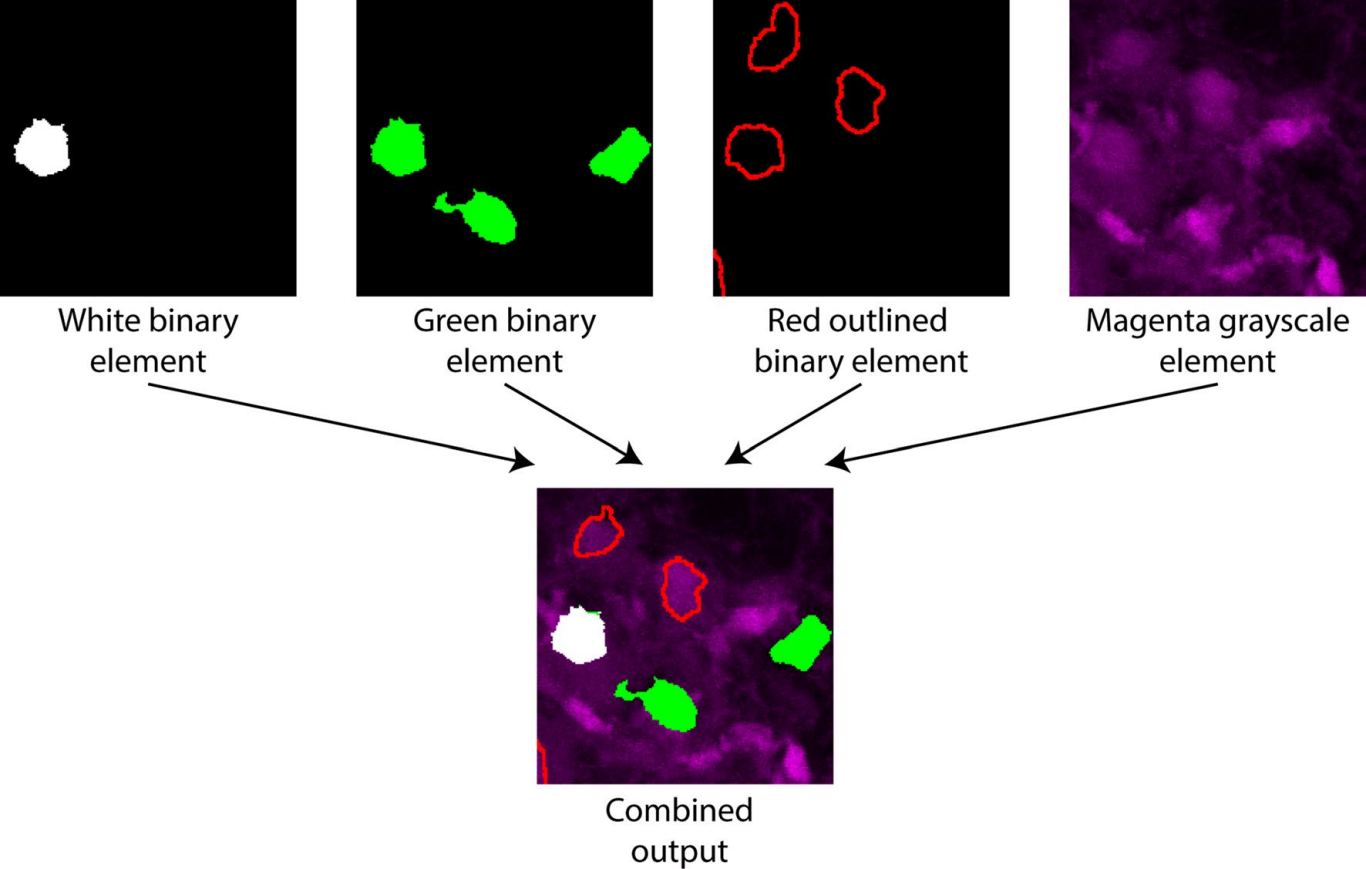
Examples of element mixing rules. The top row shows individual image elements before being combined in the output image in the bottom row. All three binary elements are prioritized over the magenta grayscale element. The white binary element has the highest color priority according to the table in (Supplementary Fig. S2 online), the green the second highest, and the red the lowest. These priorities are followed by the plugin when drawing the output image.

Note that in contrast to grayscale elements, selecting multiple input channels for a single binary element is unproblematic, and can be a very useful way to visualize object overlap between input channels, due to Boolean AND combination (see Fig. 5B—second row). This allows the user to create binary elements whose signals represent colocalization among limitless numbers of input channels. As mentioned, it is also possible to visualize colocalization *between* one binary element and other elements, including grayscale elements. This can be done by enabling the “Convert to outline” option (Fig. 5A), so that only the outlines of the binary objects are drawn (Fig. 5B—bottom row). Any colocalized pixels from subsequently added binary or grayscale elements are displayed *inside* these outlines, simulating “inspection within” the binary objects (Fig. 6). The output of a binary element can be assigned one of seven colors for display (Fig. 5A—top). This facilitates visualization of objects with distinct combinations of input channels (colocalization categories) in the output image.

Binary particles (defined by having connected pixels) of inappropriately small sizes can be removed from the output by defining a value for the “Remove connected particles smaller than” option. This can sometimes also be used to remove objects that are only partially included due to transection at a section face (Fig. 5C), since partially included objects on average should have areas smaller than completely included objects (Fig. 5C). However, this requires prior knowledge of the shape and size distribution of objects. A more robust alternative is the 3D object filter which leverages the “3D Objects Counter” plugin^12^, and can be used to exclude 3D binary objects/particles (defined by having 3D connected pixels) touching any of the XYZ edges of a Z-stack. Finally, additional input channels can be selected for the Boolean NOT operation, resulting in any signal from the NOT-selected binarized channels to be subtracted from the final output element.

Figure 5B shows the steps the Colocalization Image Creator plugin goes through when creating the binary element using the settings shown in Fig. 5A. Figure 5B also exemplifies how cell segmentation can be improved by restricting visualization to the nuclear domain of cells. Since cell nuclei are distinctly smaller than the cell somata in many cell types, restricting output signals to the nucleus often provides better separation. It is also a useful method for removing partially included objects that can give rise to potentially false negative signals, such as in the scenario shown in Fig. 2B. Restriction of output signals to the nucleus in this case would prevent an object count from being assigned to the exclusively green object to the right in Fig. 2B (containing only a cytoplasmic label), as no output signal would be produced and the object thus correctly excluded from analysis. Restricting signals to nuclei or other cellular sub-compartments is obviously also essential when analyzing cellular markers that have a compartmentalized localization (transcription factors and chromosomes are examples of potential nucleus-restricted markers).

#### Element mixing rules

Whereas grayscale elements mix colors additively if assigned to common output channels, individual binary elements do not mix or combine in any way, nor do they mix with grayscale elements. Instead, specific rules determine which element is displayed when two or more occupy the same XY (and Z) location. The rules are enforced through the plugin element processing algorithm (Fig. 3B). An overview that exemplifies these rules is shown in Fig. 6.

The first rule is that binary elements are always prioritized over grayscale elements. This means that only the signals (nonzero pixels) from the binary element will be displayed where binary and grayscale element pixels overlap. The exception is when “Convert to outline” is enabled for the binary element. In this case the outline of the binary element is still prioritized, but pixels within the outline can be from other binary or grayscale elements.

The second rule is that when two or more binary element signals occupy the same XYZ location, the conflict is resolved by checking the assigned colors of the individual binary elements. Only the binary element with the highest priority color according to the plugin’s editable “color priority table” is displayed. The default color priority goes from brightest to faintest colors, i.e. white > yellow > cyan > magenta > green > red > blue. Note that outline binary elements should be created with a color that has a higher priority than other binary elements, or they may be hidden by other binary elements or in the Z-projection. With the color priority rule, users can be certain that colored binary elements displayed in the output image are a result of the defined within-element colocalization, and not colocalization across elements. This can help keep track of what the various colors represent. However, it is usually a good idea to limit the number of image elements in a single output channel to two or three, to avoid multiple differently colored binary objects overlapping each other, which will result in showing only the one with the highest color priority. If the number of input image channels is high, it can be better to divide image elements among multiple output channels, and analyze the image by switching between the output channels (see “Examples of usage”, cases 2 and 3 below).

#### The Z-projection output

**Figure 7:**
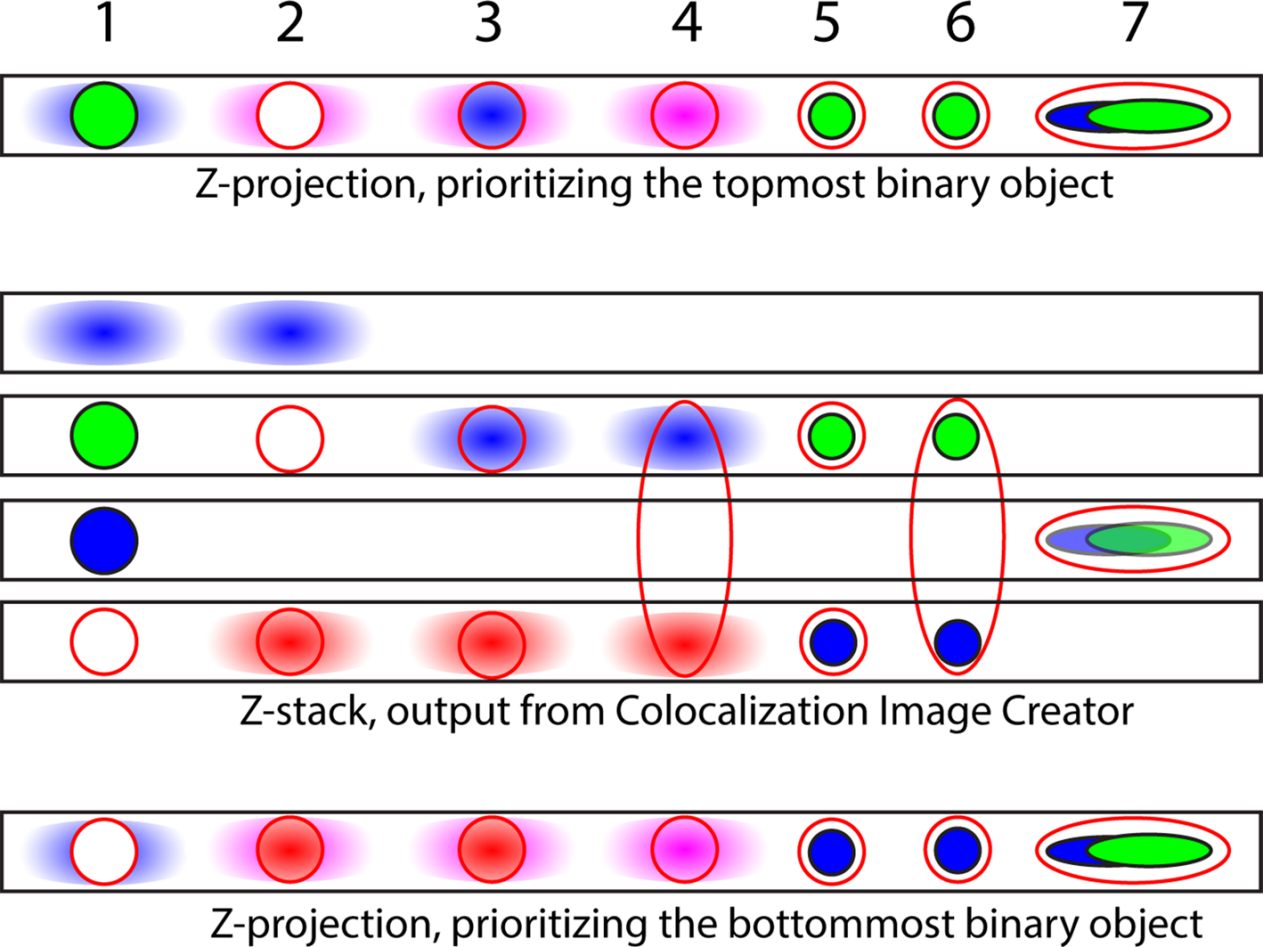
Schematic with examples of different Z-projection outputs from different scenarios of image element combinations in Z-stacks. The middle part contains four black squares that represent four slices of an output Z-stack. The Z-stack contains binary elements represented by a solid color (blue or green), grayscale elements represented colored gradient blobs (blue or red), and binary outline elements represented by red outlines. Above and below the 4-slice Z-stack are examples of Colocalization Image Creator Z-projection outputs using the top priority (above) and bottom priority (below). The numbers above refer to the seven shown example scenarios of various arrangements of image element objects, and how they affect the Z-projection outputs. For the top priority Z-projection, the explanation is as follows: (1) The topmost binary element is prioritized, green. Grayscale element pixels that do not overlap with the binary element in XY are also shown in the Z-projection. (2) The topmost binary element is prioritized, red outline. The Z-projection shows this red outline as empty since it does not overlap with any other element in XYZ. The blue and red grayscale elements mix pixels as shown in magenta in the Z-projection. (3) The topmost binary element is prioritized, red outline. The Z-projection shows this red outline containing pixels from the blue grayscale element, since it does overlap with this in XYZ. (4) The topmost binary element is prioritized, red outline, from a cell spanning three Z-sections. The Z-projection shows this red outline containing pixels mixed from the blue and red grayscale elements, since it does overlap with these in XYZ. (5) The topmost binary element is prioritized, red outline. The Z-projection shows this red outline containing pixels from the green binary element, since it does overlap with this in XYZ. (6) The topmost binary element is prioritized, red outline, from a cell spanning three Z-sections. The Z-projection shows this red outline containing pixels from the green binary element, but not from the blue binary element, since the green element is closer to the topmost Z-sections. (7) The topmost binary element is prioritized, red outline. The Z-projection shows the red outline containing pixels from the green and blue binary elements, but green is prioritized since it has higher color priority than blue in this example. Similar logic can be applied to arrive at the bottom priority Z-projection, below the 4-slice Z-projection.

In contrast to grayscale elements, binary elements represent a collection of pixels defining the presence or absence of a signal, according to the specific intensity threshold level used during element creation. This property is used by the Colocalization Image Creator plugin to avoid Z-projection artifacts (Fig. 2A) when creating output Z-projections. Z-projection artifacts are avoided by displaying only one of the image objects (for example, a cell or nucleus) when multiple image objects occupy the same XY-location, but distinct Z-locations (as the two rightmost objects do in Fig. 2A). Different relevant scenarios are illustrated in Fig. 7, which shows that users can choose to visualize either the topmost or the bottommost object in the Z-projection. Importantly, Fig. 7 also illustrates that signals from two or more grayscale input channels can produce Z-projection artifacts, whereas binary elements do not exhibit this problem due to the above mechanism.

Grayscale elements in Z-projection images are treated in the same way as within single images: binary elements always take priority over grayscale elements, except when combining grayscale elements with *outlined* binary elements (Fig. 7). Additionally, any pixels inside binary outlines in the Z-projection are guaranteed to come from where the binary element actually colocalizes with the other elements in the XYZ dimensions (Fig. 7). However, using more than one grayscale input channel can still lead to Z-projection artifacts, if the binary element object spans many Z-images, and the grayscale objects are located in multiple Z-images within the binary object outline (Fig. 7, scenario 4). This does not occur if there is a gap in the Z-axis between two separate binary outline objects (Fig. 7, scenario 3), since the two binary objects are then treated separately, and only the topmost or bottommost is shown.

The equivalent problem does not occur for binary elements within outlines, because binary elements never mix colors (Fig. 7, scenarios 5–7). Instead, either the Z-location of signals (Fig. 7, scenario 6), or the color priority (Fig. 7, scenario 7) is used for processing these.

If object classes are uniformly distributed within the volume image (without a bias to the top or bottom of the volume), and their sizes are not systematically related to specific cellular markers (such as a molecular signature), displaying only objects closest to the top or bottom edge will give an unbiased selection of objects, preserving the relative ratio of different types of binary elements that are displayed in the Z-projection image. At the same time, Z-projection artifacts are avoided. By restricting colocalization signals to a cellular compartment, such as the nucleus, the problem of object size variability can be reduced (although perhaps not eliminated, as nuclei can also vary in size). However, depending on the density of objects, and the total thickness of the original volume image, removal of Z-projection artifacts can lead to an undesired reduction of the number of objects shown in the Z-projection image, compared to the complete Z-stack (Fig. 7). This problem can be reduced by limiting the Z-depth of the input image, for example to one or two cell layers, which can be done in ImageJ using the Image > Stacks > Tools > Make Substack command. It is important to recognize that this will lead to more incompletely included objects, which can bias counting if different object classes have substantially different sizes, since large objects will be incompletely included (and thus potentially eliminated) more frequently. When the density of objects is sufficiently low, the probability of objects stacking in the Z-axis is proportionally lowered, reducing the problem of object underestimation caused by eliminating Z-projection artifacts. If an accurate absolute count of objects is desired rather than relative counts, users can instead inspect and analyze the Z-stack (see “ImageJ Plugin 2: Colocalization Object Counter” below for the quantification process), or refer to the technique of stereology, which is specifically designed for absolute quantification of object numbers^18^.

#### Verification channels

A highly recommended practice when using the colocalization image creator plugin is to add one or more “verification channels” to the output image. A verification channel is simply an input image channel added to the output image as a brightness-adjusted, but otherwise unaltered, unmixed, single grayscale element that provides an easily reviewed representation of the original image. During semi-automatic OBCA quantification (see plugin 2) users can then quickly compare binary elements with the original image data, by switching the display between the processed output image channel and the verification image channel (using keyboard shortcuts for speed). This allows rapid and effective visual verification of binary element segmentation accuracy and of colocalization in unclear cases. Verification channels are added as normal grayscale elements through the “Grayscale element menu”, by selecting a single input channel, and selecting “Create new” in the “Output image channel” box.

### ImageJ Plugin 2: Colocalization Object Counter

#### Purpose, input requirements, operation and output

The Colocalization Object Counter plugin is used for the quantification part of our OBCA pipeline, and also comes with a set of tools for subsequent 3D reconstruction of data. It enables semi-automatic identification, quantification and XYZ-coordinate designation of image objects, and comes with a set of effective and simple tools for assigning specific *colocalization categories* for each object (associating specific cellular markers or labels with objects). For automatic object detection, it uses local maxima algorithms to place ImageJ multipoints (XYZ-coordinate points) to define objects, which can be edited and verified visually in a semi-automatic manner. The user can choose between having multipoints assigned and displayed only on specific Z-images, or all Z-images, depending for example on whether a complete Z-stack is analyzed in detail, or if only a Z-projection of the stack is analyzed. The highest accuracy and speed are normally achieved when automatic detection is followed by manual verification and adjustment.

Any image readable by ImageJ can be used with the plugin, including those generated with the Colocalization Image Creator plugin. The plugin supports assignment of up to eight different categories per object, allowing for 2^8^ = 256 possible colocalization combinations. See the “Supplementary Information” section online entitled “ImageJ plugin 2” for specific details on the plugin operation and user interfaces.

Following assignment of objects and categories, data can be saved as files (comma separated value format, CSV) that include the XYZ-coordinates and the colocalization categories of each object, the image filename and additional image metadata. Colocalization categories are specified by a string of digits; for example, “358” designates an object that is positive for categories 3, 5 and 8. In a typical experiment the category “1” would be used to designate all objects of interest, and additional category designations would be used to annotate the presence of additional markers (such as fluorescent labels) within the objects. This of course requires a generic marker for the objects of interest, such as a general cellular or nuclear marker. To help the user organize the data, all the output files are saved automatically in the same folder from which the image was loaded, in a subfolder called “Counts”. It is therefore highly recommended to store all images from the same experimental series in a single folder. Additional output files are generated in the same “Counts” folder after using the 3D serial reconstruction tools included with the Colocalization Object Counter plugin, which includes a tool for assigning the image origin coordinates and north direction, and a tool for drawing tissue contours (see below). Together with the object count data, these can be imported and analyzed with the Excel macro file (see below), and subsequently visualized in 3D with the MATLAB script (see below), both of which are automatically supplied and placed by the plugin in the appropriate output folders. To facilitate logging of operator interventions for later review, all operations involving object counting are automatically logged in a file named “Counting_log.txt” in the “Counts” folder.

#### Manual counting

**Figure 8:**
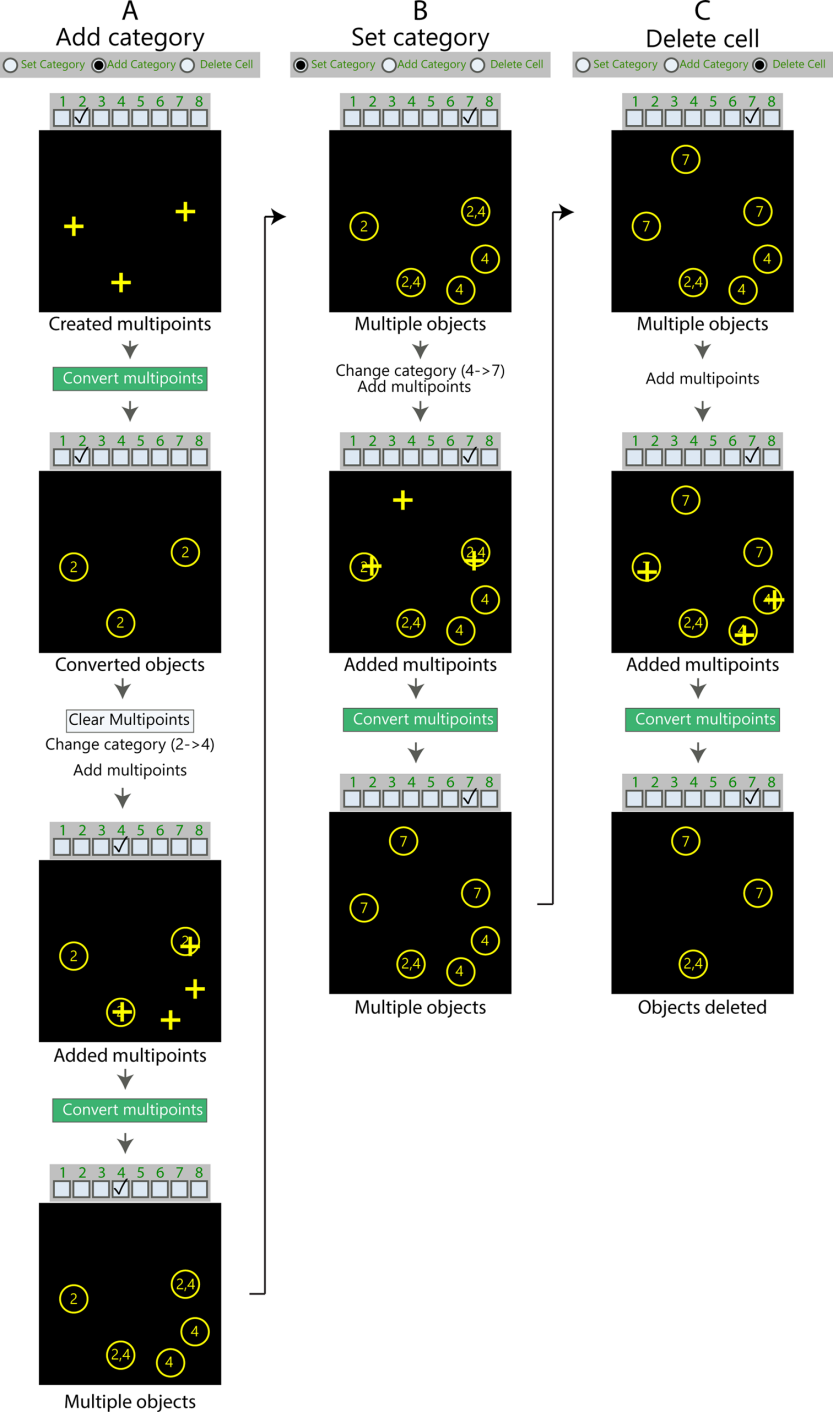
Examples of how to use the three counting modes in the Colocalization Object Counter plugin. (**A**–**C**) Step-by-step flowcharts illustrating how to use the “Add category” mode (**A**), the “Set category” mode (**B**), and the “Delete cell” mode (**C**). For the “Add category” and “Set category” mode, multipoints are marked and converted to circular overlays by clicking the “Convert multipoints” button. Selected categories are respectively added or set for existing overlays. For the “Delete cell” mode, overlays are deleted by marking them with multipoints and clicking “Convert to multipoint”. See main text for detailed description of the individual panels.

During manual operation of the Colocalization Object Counter plugin, multipoint markers are manually placed by the user over identified image objects. An appropriate colocalization category value is chosen and the multipoints are converted to circular overlays representing each image object, with the overlay names displayed according to their colocalization category value (Fig. 8). Additional colocalization categories can subsequently be assigned to subsets of the overlays by changing the active colocalization category value, and placing new multipoints *inside* the circular overlays of appropriate objects. See the “Supplementary Information” section online entitled “Manual counting” for additional details on the counting procedure.

#### Using circular object overlays to define colocalization in a centroid-based approach

Since detected objects are represented with circular overlays, and further colocalization assignments to the objects are determined by whether subsequent markers fall inside or outside the overlays, we have used a centroid-based approach to colocalization. The radius of the circular overlays can be changed by adjusting “overlay size” in the menu, thereby changing the threshold for the required distance for assigning colocalization between an overlay and a newly placed marker. For automatic detection of objects, increasing the “pre-blur” setting for the “Find Maxima” process tends to result in markers being placed at the center of objects. One of the benefits of a centroid-based approach is that colocalization can be assigned for markers that are in close proximity but do not have overlapping pixels. This can be desired for example when using markers that have exclusive and highly regional domains (nuclear, cytosolic, extracellular matrix), allowing these to be assigned to a single cell regardless of the degree of pixel overlap.

#### Automatic object detection

The plugin has two built-in tools for automatic object detection; one for single 2D images, and one for 3D images (Z-stacks). The 2D tool utilizes the built-in ImageJ function “find maxima”, and the 3D tool utilizes the “3D Image Suite–3D maxima finder” plugin^19^. Detection is activated by pressing the “Find 2D/3D maxima” button in the “Automatic Detection” panel (Supplementary Fig. S4 online). This results in the automatic placement of multipoint markers over detected objects. During semi-automatic operation, inaccurately placed markers can be adjusted manually by moving the markers by mouse dragging, by deleting markers with alt + left-clicking, or by adding more markers by left clicking the mouse as during manual operation. Following detection and adjustment, the created multipoints are converted to overlays in the same way as for manual counting.

The “Automatic detection” tool-panel contains the following options: (1) “Noise tolerance” sets the sensitivity level of detection. (2) “Pre blur radius” applies a Gaussian blur of specified radius before detection. This can be useful to avoid creation of several multipoints on the same object, or to center multipoints to the middle of objects. (3) “Exclude on edges” ignores edges. (4) “Light background” assumes a light image background. (5) “Color to detect” restricts the tool to detect objects of the selected color. (6) “Only inside outlines” is intended for use with images from the Colocalization Image Creator plugin. Selecting a color here will restrict detection to within outlines of the selected color. (7) “Radius xy(z)” are detection settings for the 3D maxima. (8) “Show filtered output” shows the output of the performed image filtering operations.

Advanced users can develop their own object detection algorithms using custom ImageJ macros or plugins for multipoint creation. These can thus be combined with the basic functions of the Colocalization Object Counter plugin. Custom macros or plugins need only to output multipoint markers from the active image.

#### Serial 3D reconstruction of objects and tissue contours

The Colocalization Object Counter plugin comes with two tools that are used in connection with 3D reconstruction of objects and tissue contours from image series. One tool assigns a reference origin coordinate and the “north” direction of images, and the other is used for drawing tissue (or other) contours. Data collected with these tools are automatically saved to CSV files, and used in preparing and visualizing the 3D data using the Excel macro file and the MATLAB script described below.

### Excel macro file for data organization

#### Purpose, input requirements, operation and output

The purpose of the Excel macro file (Supplementary Fig. S5 online) is to import data created with the Colocalization Object Counter plugin, assign Z-axis levels to the images, translate XY coordinates according to reference origin and north, edit data points if needed, summarize and display counting data, and export the combined data in files for subsequent 3D reconstruction in MATLAB or other programs. The Excel file (“ColocalizationObjectCounter_Importer.xlsm”) is automatically placed by the Colocalization Object Counter plugin in the “Counts” folder for the respective image series, from where it will be able to automatically locate and read any data files it needs. All the input and output data files are stored in CSV format. After opening the Excel file, click “Enable macros” if a security warning is displayed. See the “Supplementary Information” section online entitled “Excel macro file for data organization” for details on the macro file operation and user interfaces.

### MATLAB script for 3D visualization

#### Purpose, input requirements, operation and output

The purpose of the MATLAB script is to generate and visualize an interactive 3D reconstruction of object and contour data, with the different colocalization categories of objects visualized in separate colors. The 3D figure can be rotated, zoomed, panned and animated. The script requires appropriate input CSV files containing object and contour data, which are generated by the Excel macro file described above. A graphical version of the script launches a simple graphical menu, from which the 3D reconstruction can be generated (Supplementary Fig. S6 online). An additional version without a graphical menu that is compatible with MATLAB versions older than R2018a (9.4) is also available, in which display options have to be set by editing the .m file in a text editor. All MATLAB script files required for operation is automatically placed by the Colocalization Object Counter plugin to the images “/Counts/Export” folder. See the “Supplementary Information” section online entitled “MATLAB script for 3D visualization” for details on the macro file operation and user interfaces.

## Examples of usage

To illustrate these tools in action, we present here three examples of usage taken from our own research on the molecular determinants of brainstem-to-spinal cord projection neurons^20^. Examples 1 and 2 illustrate different uses of the Colocalization Image Creator, whereas example 3 illustrates use of our complete analysis pipeline, which includes all four plugins and scripts. We point out that our material has been sectioned at 20 μm to promote full penetration for immunohistochemical staining, but that the analysis platform can be used with thicker sections or even unsectioned tissue that is appropriately cleared and contains labeled objects^21^.

**Figure 9:**
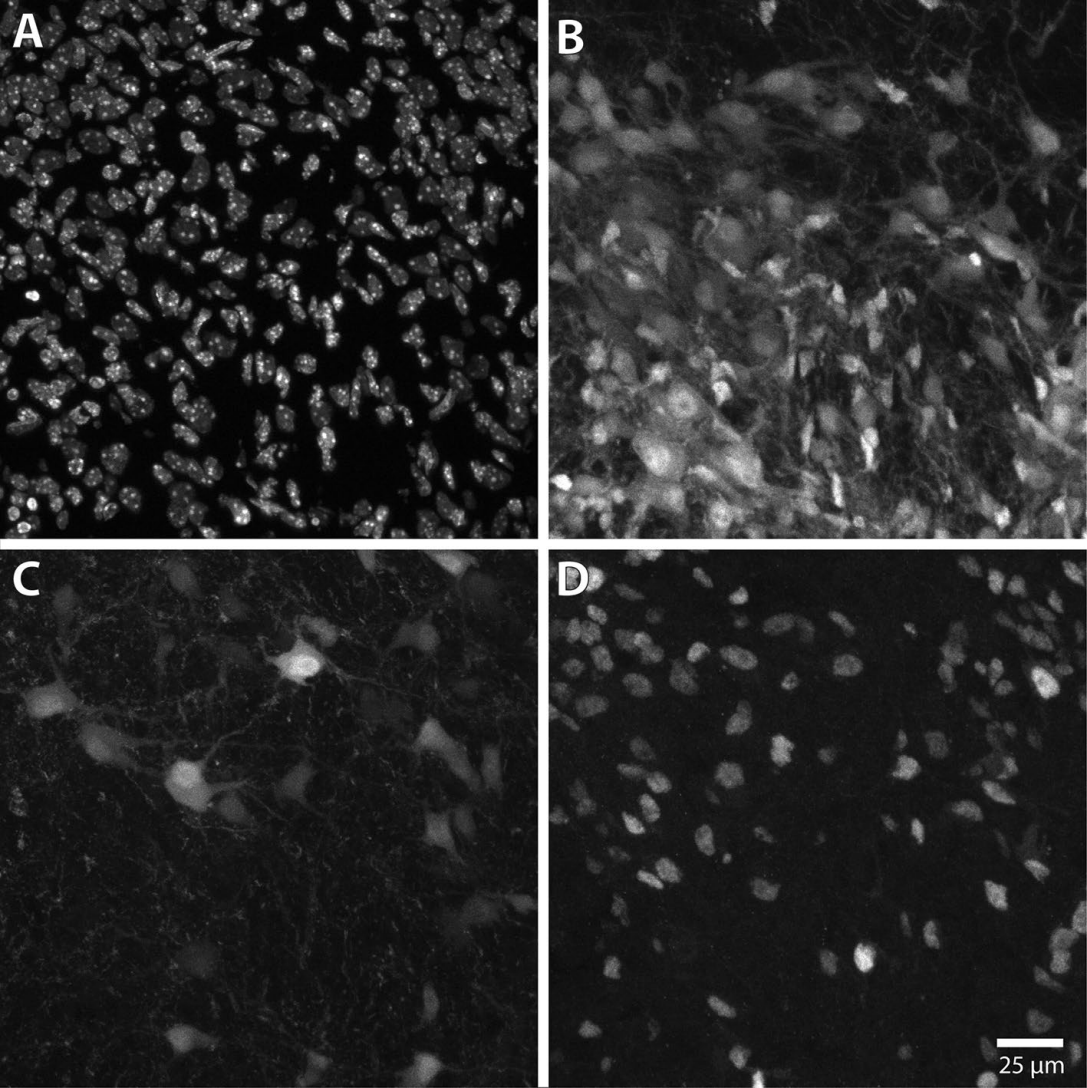
Maximum intensity Z-projections of input image channels used in examples 1 and 2. The panels show brightness adjusted maximum intensity Z-projections of the confocal Z-stack image used in Examples 1 and 2. The image is of a 20 μm thick transverse cryosection taken from the brain stem of a prenatal mouse (see “Materials and methods”). (**A**) Channel 1, showing all nuclei stained with Hoechst 33258. (**B**) Channel 2, showing fluorescent protein expression (tdTomato) in all neurons that have expressed Cre. The tdTomato label is distributed throughout the cytoplasm. (**C**) Channel 3 showing retrograde labeling with a fluorescein-conjugated dextran amine applied to the cervical spinal cord. The fluorescence label is distributed throughout the cytoplasm. (**D**) Channel 4, showing immunostaining for the nuclear protein Lbx1.

The input image in examples 1 and 2 is a confocal Z-stack of a small area of a cryosection of mouse brainstem, with four image channels showing respectively the following fluorescent labels: nuclear staining with the dye Hoechst 33258, tdTomato expression activated in a population of Cre-expressing neurons, neurons retrogradely labeled from the spinal cord using fluorescein–conjugated dextran amines, and immunohistochemical labeling of the nuclear protein Lbx1 (Fig. 9). The image consists of 35 optical slices of 1 μm thickness taken at 0.48 μm intervals (in accord with the Abbe diffraction limit and the Whittaker–Nyquist–Kotelnikov–Shannon sampling theorem), from a 20 μm thick tissue section.

Example 3 uses three images from the same mouse brain stem with the same fluorescent labels, but are tile-scanned confocal Z-stacks of whole transverse brainstem sections. These show retrogradely labeled neurons on one side of the brain stem. They each consist of 28–33 optical slices of 1 μm thickness taken at 0.48 μm intervals, from a 20 μm thick tissue section.

See “Data availability” for access to the example images and Colocalization Image Creator settings files.

### Example 1: Visualizing colocalization between two neuronal cell populations using the Colocalization Image Creator

**Figure 10:**
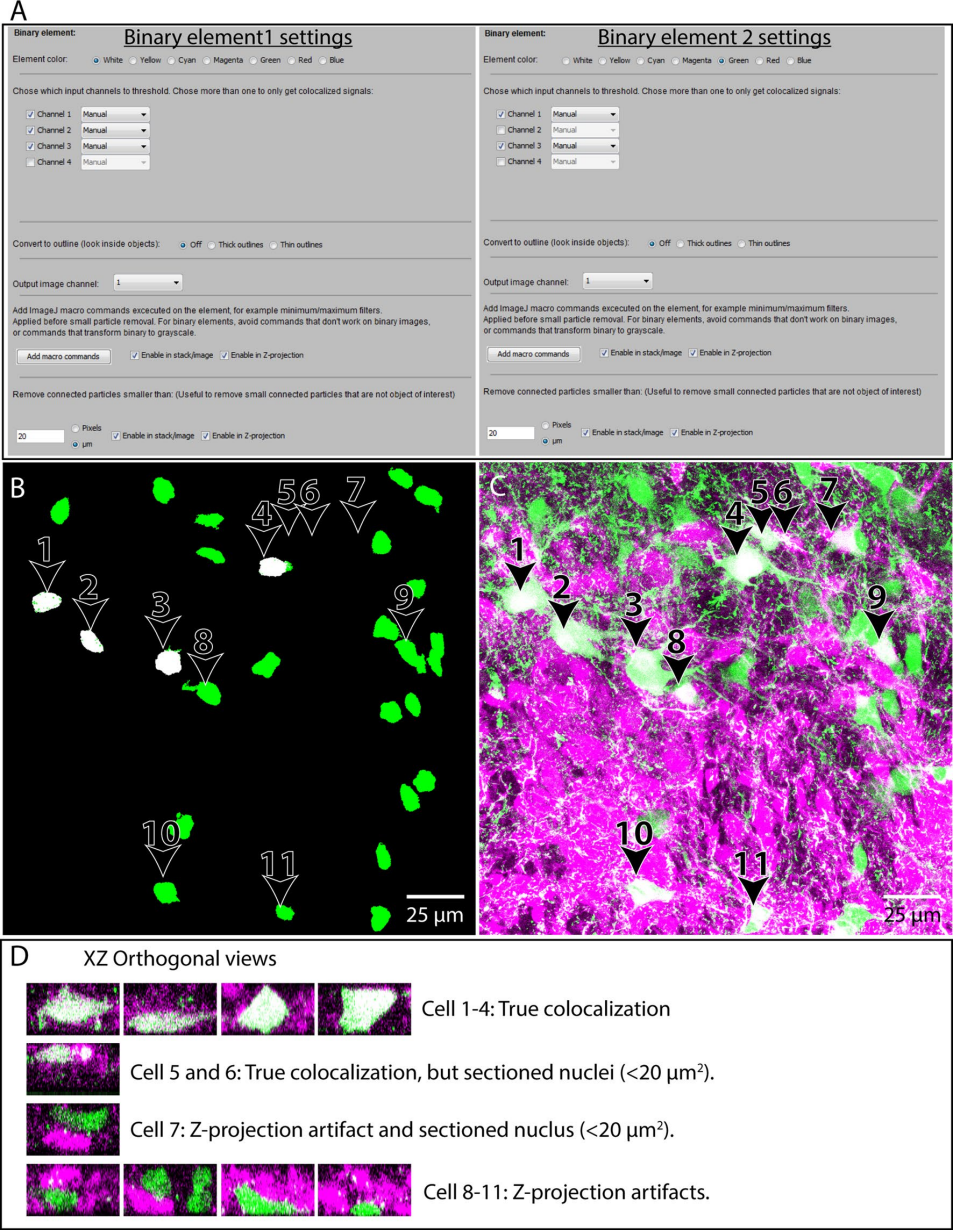
Example of usage 1. Visualizing Colocalization between two neuronal populations using the Colocalization Image Creator. Overview of the element settings (**A**) used with the Colocalization Image Creator plugin, and the Z-projection output (**B**). Three channels of the input image (see Fig. 9) are used: channel 1 showing nuclear staining (Hoechst 33258), channel 2 showing neurons labeled with tdTomato expression, and channel 3 showing retrogradely labeled neurons. (**B**) Z-projection output using the element settings in (**A**). White objects represent colocalization of nuclear, retrograde, and tdTomato labeling. Green objects represent colocalization of nuclear and retrograde labeling only. Arrowheads and numbers in (**B**) indicate the neurons shown in (**C**,**D**). (**C**) Standard maximum intensity Z-projection of the grayscale data of the two neuronal populations, using the built-in ImageJ tool (Stacks- > Zproject- > Maximum). Retrogradely labeled neurons are green, tdTomato-expressing neurons are magenta. White pixels indicate overlap, either due to true colocalization or Z-projection artifacts. (**D**) XZ orthogonal views of the neurons indicated in (**B**,**C**), which are colored as in (**C**). The different rows show examples of true colocalization, Z-projection artifacts, and neurons whose nuclei have been transected during tissue sectioning. Note how only neurons 1–4 have true colocalization of signal in all three input channels, and are completely included in the section, which is clearly shown in (**B**). Note also that the images in (**C**, **D**) are not outputs of the Colocalization Image Creator plugin, but only presented to evaluate the efficiency of the Z-projection artifact removal.

In this example we want to compare a population of neurons retrogradely labeled with a fluorescent tracer to a population of neurons expressing a fluorescent reporter protein, and to determine which neurons are labeled with both markers, and which are only retrogradely labeled. To do this, we will use two binary image elements: one to represent colocalization of retrograde labeling and nuclei, and another to represent colocalization of retrograde labeling, reporter expression and nuclei (Fig. 10). Although not strictly necessary, for purposes of clarity in the example we restrict visualization of colocalization signals to the nuclei of the neurons, since neuronal somata in close proximity can be difficult to distinguish. This is recommended whenever an image channel of nuclear staining is included in the image data. Although not included in this example, it can be helpful to output the neuronal somata as grayscale elements/verification channels in addition, to help discriminate abutting neurons.

We choose the binary image element representing retrograde/reporter/nuclear colocalization to be colored white, and the binary image element representing retrograde/nuclear colocalization to be colored green. Since the binary color priority of white is higher than green by default, white elements are automatically “overwriting” green elements. However, this does not hide any objects per se, since retrograde/reporter/nuclear objects (white) is a *colocalization combination superset* of retrograde/nuclear objects (green). In other words, all triple-labeled neurons are by necessity also double-labeled.

To remove signals from axons, dendrites, cell borders, and to remove some background, we add the “Minimum filter” ImageJ macro command with a radius = 1 pixel, and the “Fill holes” command to both binary image elements. We also remove particles smaller than 20 μm^2^ (2 SDs smaller than the mean of complete nuclei profiles—determined empirically for the data set), which generally eliminates nuclei that are only partially included within a section (see Fig. 5C). The settings for the image elements are shown in Fig. 10A.

The generated output Z-projection (Fig. 10B) removes several Z-projection artifacts, and only shows truly triple-labeled neurons as white (in this case only their nuclei, which are separated and easily distinguished). By comparison with a standard grayscale Z-projection (Fig. 10C), and a XY-orthogonal view of neurons, it is clear that several cases of Z-projection artifacts and partially included nuclei have been removed.

### Example 2: Visualizing colocalization of immunostaining, retrograde labeling and reporter protein expression using the Colocalization Image Creator

**Figure 11:**
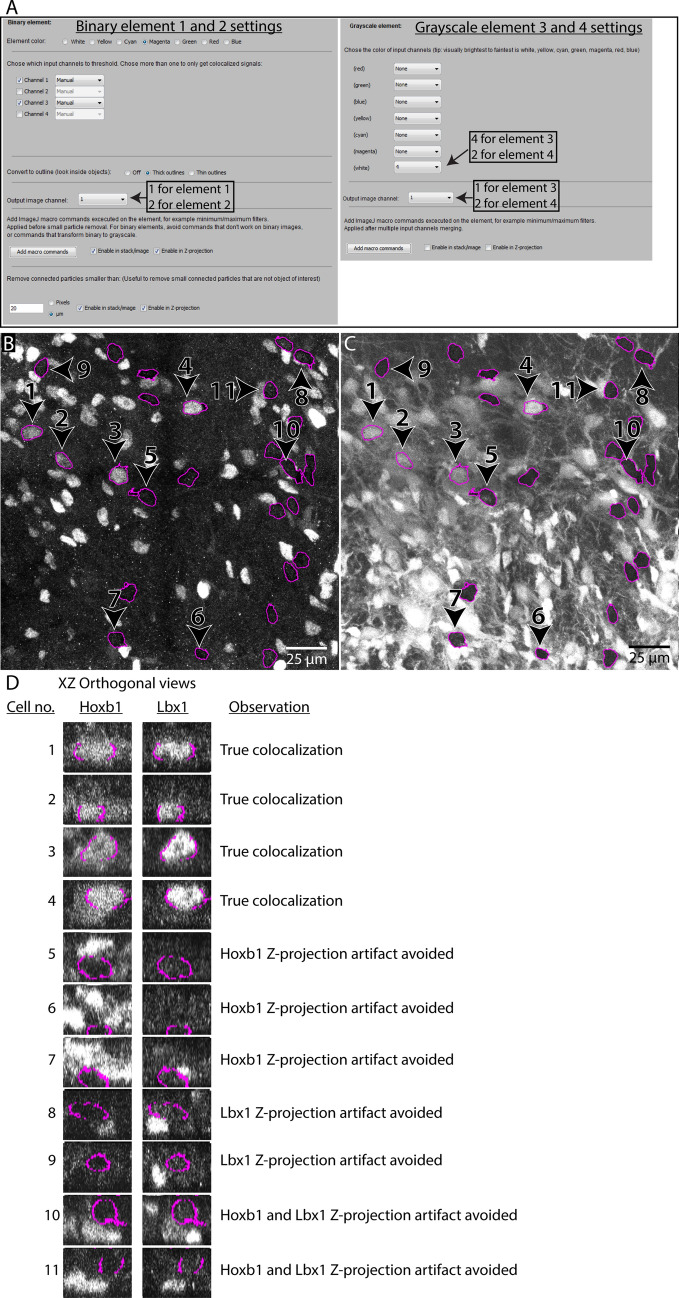
Example of usage 2. Visualizing colocalization between immunostaining, retrogradely labeled neurons, and reporter protein expression using the Colocalization Image Creator (**A**) Overview of the element settings used with the Colocalization Image Creator plugin to create the output shown in (**B**,**C**). Four channels of the input image (see Fig. 9) are used: channel 1 showing nuclear staining (Hoechst 33258), channel 2 showing neurons expressing the tdTomato reporter protein, channel 3 showing retrogradely labeled neurons, and channel 4 showing immunostaining for the transcription factor Lbx1. (**B**) The Z-projection output of channel 1 of the output image, comprising elements 1 and 3, visualizing respectively outlines of the nuclei of retrogradely labeled neurons (magenta) and Lbx1 immunostaining (white). (**C**) The Z-projection output of channel 2 of the output image, comprising elements 2 and 4, visualizing respectively outlines of the nuclei of retrogradely labeled neurons (magenta) and tdTomato reporter expression (white). (**D**) XZ orthogonal views of neurons from the output image stacks in output channel 1 (left column) and output channel 2 (right column). These images are not part of the plugin output, but are shown to aid in evaluating the efficiency of Z-projection artifact removal. Neurons are numbered as indicated in (**B**,**C**). Different rows show examples of true colocalization and examples of Z-projection artifacts in each output channel.

In this example, we want to visualize colocalization between immunohistochemical staining for a nuclear protein (Lbx1) and retrogradely labeling, and compare it to the expression of a fluorescent reporter protein (tdTomato) (Fig. 11). We use the same input image as in Example 1, using this time all four input channels. Since Lbx1 protein is restricted to the nucleus, it is critical to restrict visualization to only the nuclei of retrogradely labeled neurons. Otherwise, false negative counts can arise from neuronal somata lacking nuclei, due to incomplete inclusion within a tissue section (see Fig. 2B). Moreover, many staining methods, including immunohistochemical staining, may have a low signal to noise ratio, making it difficult to segment effectively by thresholding. We will therefore visualize the anti-Lbx1 and the tdTomato signals as grayscale elements. We output the grayscale elements to separate output channels to avoid visualization errors that can occur when combining grayscale elements. To represent the nuclei of retrogradely labeled neurons, we create binary elements as in Example 1, but with the “Convert to outline” option set to “Thick outlines”.

We first generate the two binary outline elements, one for each of the two output channels, but otherwise identical. The settings are the same as for element 2 in Example 1, except that the “Thick outlines” box is checked and the color magenta is chosen instead of green. Next, we add the anti-Lbx1 signal as a white grayscale element in output channel 1, and the tdTomato signal as another white grayscale element in output channel 2. The settings for the image elements are shown in Fig. 11A. Finally, we add two verification channels that can be used to verify the binary segmentation behind the magenta outlines as necessary. These are added as a white grayscale element showing the raw signal from the retrogradely labeled neurons (as seen in Fig. 9C), which we assign to output channel 3, and as a white grayscale element showing the raw signal from the nuclear staining (as seen in Fig. 9A), which we assign to output channel 4.

Since we will use the image element settings created here again for multiple images in Example 3, we save the settings to a file by clicking the “save settings” button in the main menu (“example2_settings.json”).

The resulting output Z-projections (Fig. 11B,C) are devoid of Z-projection artifacts and false negatives due to incompletely included nuclei, and clearly show which retrogradely labeled neurons express Lbx1 or tdTomato (compare with Fig. 11D). Switching between channels 1 and 2 using the keyboard shortcuts Q and W facilitates the comparison of Lbx1 and tdTomato labeling, and shows that Lbx1 signal (Fig. 11B) is only present in neurons that also contain tdTomato signal (Fig. 11C). The validity of the magenta outlines can be verified by comparing channel 2 (keyboard shortcut W) with the verification channels in channel 3 and 4 (keyboard shortcut E and A).

### Example 3: Working with large images, batch processing, semi-automatic colocalization quantification and 3D reconstruction

**Figure 12:**
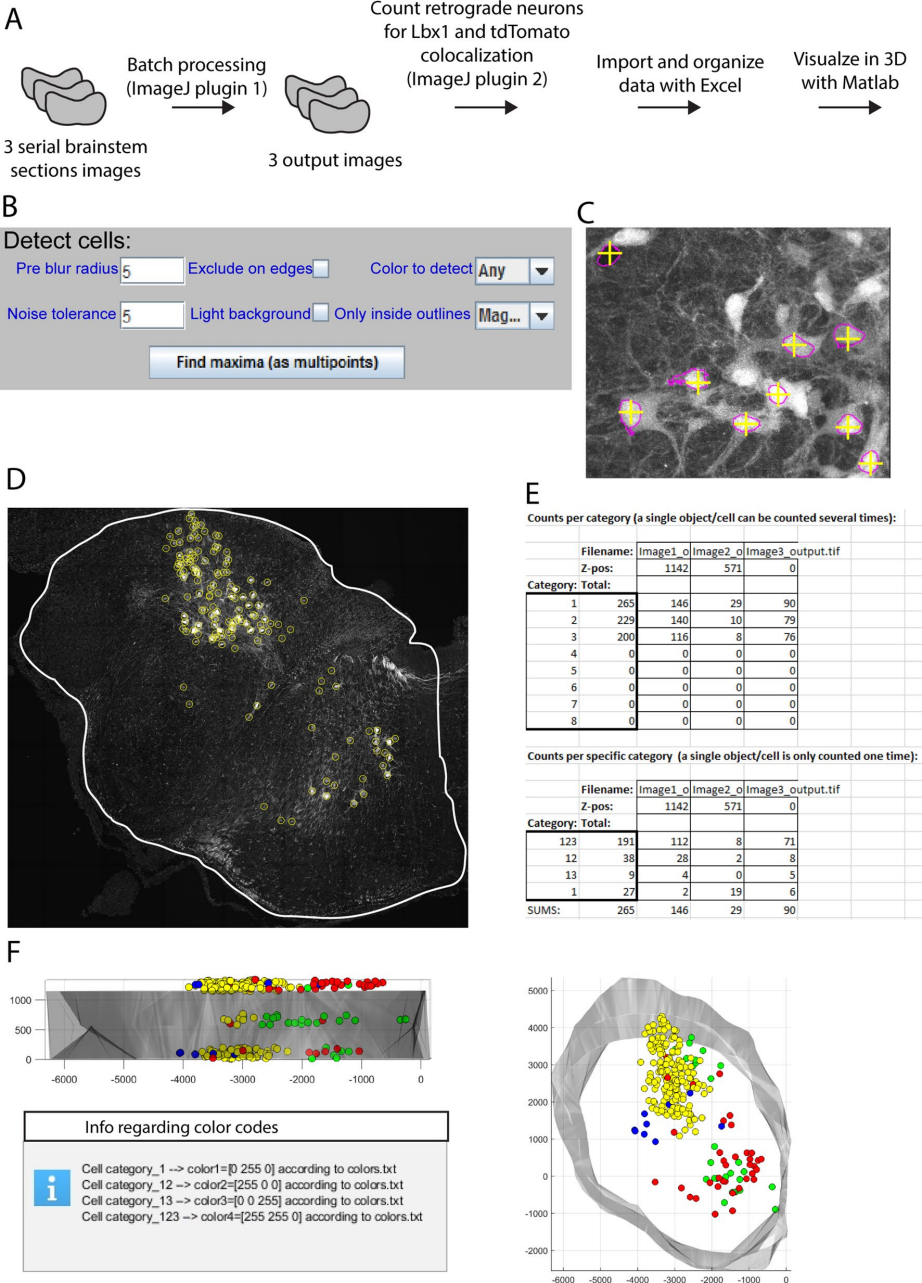
Example of usage 3. Working with large images, batch processing, semi-automatic colocalization quantification and 3D reconstruction. (**A**) Overview of the workflow. The input comprises 3 image Z-stacks of transverse sections through the left half of a mouse embryo brain stem. The imaging channels and fluorescence labels are the same as in examples 1 and 2. (**B**) Settings used for automatic detection of all nuclei of retrogradely labeled neurons in output channel 2. (**C**) Automatically marked magenta outlines, using the parameters shown in (**B**). Note the high accuracy of the multipoint markers. (**D**) A whole brainstem section after conversion of multipoints to overlays, representing all the nuclei of retrogradely labeled neurons. (**E**) Summary of the object count data after importing into the Excel macro file. (**F**) The 3D reconstruction of the data processed in the previous steps, seen in the transverse plane (right) and from the side (top left). The message box at bottom left indicates the relationship between the point colors and object categories.

In this example we will apply the element settings from Example 2 to three large Z-stack images of serial transverse sections using batch processing. We will then semi-automatically quantify the different colocalization categories of retrogradely labeled neurons using the Colocalization Object Counter plugin, import and organize the data with the Excel macro file, and visualize the combined data in a 3D reconstruction using the MATLAB script. The workflow is summarized in Fig. 12A. For tips and details on operations and user interfaces related to this example, see the “Supplementary Information” sections online entitled “Working with large images”, and “Notes on reusing, adjusting, and verifying threshold and brightness level settings for batch processing”.

#### Batch processing

In the batch processing menu we click “Load element profile” to load the settings file from Example 2 (“example2_settings.json”), and specify the input image folder and the output folder. We click “Start batch processing”, and monitor the progress in the ImageJ log window. Each input image is about 11 GB in size, takes about 45 min to process with the example element settings, and has a peak memory consumption of about 110 GB.

#### Semi-automatic OBCA with the Colocalization Object Counter plugin

After processing the images, we open them one by one in ImageJ and start the Colocalization Object Counter plugin. We start by marking multipoints on the nuclei of all the retrogradely labeled neurons. These are indicated with magenta outlines in channel 1 and 2. Since there are over a hundred such neurons in each image, we can speed up this process by using the plugin’s automatic detection tool. In this case, we can detect the signals (background or strongly labeled) inside all magenta outlines in channel 2 to automatically mark them. We set a low “noise level” of 5 to include detection of background signals, and a “pre-blur” to 5 to bias multipoints to the center of the outlines. We set “Only inside outlines” to “magenta” to restrict detection to within magenta outlines. These settings are shown in Fig. 12B. Finally, we click “Find maxima” which results in automatic multipoint marking. We then manually verify that the automatically marked outlines are accurate (Fig. 12C) by comparing outlines in channel 2 with raw retrograde signal in channel 3 and raw nuclear staining in channel 4. Using keyboard shortcuts enables quick verification and correction of any mistakes made by the automatic detection. After removing a few multipoints that were falsely assigned due to instances of retrogradely labeled axons overlapping labeled nuclei in the Z-axis, we click the green button labeled “Convert multipoints to cells”. This converts the multipoints into persistent circular overlays each with a number in the middle that indicates its colocalization category (Fig. 12D).

Now that we have marked all retrogradely labeled neurons with persistent overlays, we want to identify which ones are positive for both, none, or either of the other two labels. Still in channel 2, which visualizes the tdTomato signal inside the retrogradely labeled neurons, we change the colocalization category to 2 (near top of the main menu), and clear previous multipoints by clicking “clear multipoints”. By changing the active category, new multipoints created inside already existing overlays will indicate colocalization with the selected category (tdTomato). Again, we use automatic detection to speed up this process. We set “Pre-blur radius” to 2, “Noise tolerance” to 60, and “Only inside outlines” to magenta, and click “Find maxima”. To remove multipoints that are created outside of overlays generated in the first step we click the “Toggle multipoints inside/outside/all cells” once. We inspect the automatic detection as before, removing a few unintended multipoints generated by weak signals, add some that didn’t discriminate merged cells, and click “Convert multipoints to cells”.

Finally, we change to channel 1, which visualizes the Lbx1 signal, and change the active colocalization category from 2 to 3. We change “Noise tolerance” to 150, click “Find maxima”, toggle once to hide multipoints marked outside of overlays generated in the prior two steps, and verify markers. Finally, we click “Convert multipoints to cells”. We now have the counts, coordinates, and categories of all retrogradely labeled neurons, according to colocalization with either tdTomato, Lbx1, or both. To save these data to a file, we click “Save counts”.

#### Indicating origin and north direction and drawing contours for 3D reconstructions

To generate a 3D model with the MATLAB script, we also draw the contour around the tissue, indicate the coordinate origin (x = 0, y = 0), and the “north” direction of the image, using the appropriate buttons in the Colocalization Object Counter plugin. See the “Supplementary Information” sections online entitled “Serial 3D reconstruction of objects and tissue contours” for details on this operation. We repeat the process for the two other images, clicking “Reset all sets/counts” between images.

#### Importing and organizing data with the Excel macro

The above steps automatically save the data files for cell counts and 3D reconstruction to the “Counts” folder, which is located in the same folder as the image files. To import and summarize the data, we open the “ColocalizationObjectCounter_Importer.xlsm” file, and click “Enable Content” in the macro security warning. In the image series, we have analyzed every 6th serial section of 20 μm thickness, giving us a value of 6 × 20 μm = 120 μm Z-level distance between sections. Columns I and J tell us that the XY-unit of our input images is 0.21 μm/pixel.120 μm/0.21 μm/pixel = 571 pixels.

We therefore write “571” in cell D5, “yes” in cell D6, and click button 1. For step 2, we add some Z-level jitter to each object (to “randomize” positions so they do not line up strictly with section planes) by writing “190” (571 pixels/3 = 190 pixels) in cell D22, click button 2, and thereafter button 3. This brings us to the “Data summary” sheet, which shows us the total and per section counts for each of our categories (Fig. 12E).

#### 3D visualization with MATLAB

By clicking button 3 in the Excel macro, we automatically generated 3D visualization data files in the “\Counts\Export” folder. We navigate to that folder and run the MATLAB .m file. We adjust the scatterpoint size to 30, and alpha value to 1200, and click “Visualize”. In the newly created window we can clearly see the contours of the tissue as the alphashape, and the retrogradely labeled neurons as scatterpoints. Clusters of different categories of retrogradely labeled neurons are clearly seen on the ipsilateral (same side) and contralateral (opposite side) (Fig. 12F). Information regarding color codes is displayed in a message box.

### Assessment of output consistency across users of semi-automated quantification

To assess the consistency of output across different users employing semi-automatic OBCA with the Colocalization Object Counter plugin on image output from the Colocalization Image Creator plugin, we recruited five different naïve users to independently analyze the three images used in Example 3 (Table 1). Their results were compared to ground truth values, determined by an expert, and to values obtained from the plugin’s fully automated detection capabilities, using detection parameters assigned by an expert. The results showed that even with minimal training in using the plugin, results were highly consistent, with accurate colocalization assignments ranging from 95 to 99%. The semi-automatic quantification was substantially more accurate than the fully automated quantification (80%). This shows that semi-automatic OBCA using our tools provides accurate and consistent results across different users.

**Table 1:** Assessment of output consistency across users of semi-automated quantification, compared with fully automated methods.

	Ground truth	CellProfiler	Automatic	User 1	User 2	User 3	User 4	User 5
Non-target cells (not counted)	Thousands (nc)	nc	nc	nc	nc	nc	nc	nc
Correctly assigned target cell and colocalized markers	284	195	223	272	265	272	264	266
Correctly assigned target cell, incorrect colocalized markers	0	13	48	1	4	1	9	8
Non-target cell assigned as target cell	0	36	8	3	4	3	6	2
% Accurate assignments	100.0%	79.9%	79.9%	98.6%	97.1%	98.6%	94.6%	96.4%
Target cell not assigned (omitted)	0	76	13	11	15	11	11	10
% Target cells not assigned (omitted)	0.0%	26.8%	4.6%	3.9%	5.3%	3.9%	3.9%	3.5%

The table shows quantification results of the three images used in Example 3 generated by an expert (ground truth), a CellProfiler^22^ pipeline (see methods), automated counts with our plugin, and five independent users. Target cells represent all retrogradely labeled neurons containing colocalized nuclear staining, which were assessed for the tdTomato marker activated in a population of Hoxb1Cre-expressing neurons, and immunohistochemical labeling of the nuclear protein Lbx1. Ground truth distribution of markers in target cells were 29 Lbx1−/Hoxb1− neurons, 9 Lbx1+/Hoxb1− neurons, 34 Lbx1−/Hoxb1+ neurons, and 212 Lbx1+/Hoxb1+ neurons. Parameters for 2D automatic counting with the Colocalization Object Counter plugin were for (a) target cells: noise level 1, blur radius 8, only inside magenta, (b) Lbx1: noise level 63, blur radius 8, only inside magenta, (c) tDTomato: noise level 63, blur radius 8, only inside magenta.

### Comparison to an existing automated colocalization analysis platform

Having shown that using our platform in fully automated mode gave substantially less accurate results than using it in semi-automatic mode, we decided to make an additional comparison to a fully automated analysis pipeline with the widely used CellProfiler software^22^. As with the fully automated mode in our platform, the fully automated mode in CellProfiler gave substantially lower accuracy (80%, Table 1) than our platform in semi-automatic mode.

## Discussion

The expression of distinct sets of genes is a defining characteristic of different cell types and sub-types^1,2^. Analysis of this molecular diversity in a spatial context, particularly at the protein level, can be performed through differential fluorescence labeling and multi-fluorescence imaging, followed by colocalization analysis^3,12,13,23^. Many available colocalization analysis tools and algorithms are designed to function fully automatically. This is great for speed and objectivity, but can compromise accuracy, as witnessed by our assessment of output consistency, for which automated counting was substantially less accurate than semi-automated counting. Pixel-based colocalization algorithms do not facilitate the definition of object categories. Object-based colocalization analysis (OBCA) is usually preferred because it defines objects, but most OBCA platforms require high fidelity object segmentation. This can be difficult to achieve automatically, and time-consuming to do manually.

There is a general lack of tools, algorithms, and workflows for semi-automatic OBCA that enable cell-by-cell assessment using multiple labels and that rely less on automatic object segmentation. Development of such tools can help remove the image-analysis bottleneck in experimental pipelines that involve complex image data sets. This would facilitate more ambitious experimental designs and more efficient and accurate processing of complex data. In our experience, such tools would be particularly valuable for analyzing the molecular diversity of cell types in the developing central nervous system (CNS), but they would be generally applicable to other tissues as well.

To create a useful OBCA platform for our own studies of the molecular diversity of neurons in the embryonic CNS^20^, we developed the four tools presented here, designed to work together in a streamlined OBCA workflow. We developed two ImageJ plugins that (1) process multi-label imaging data into a visual format that is more suitable for semi-automatic OBCA (the Colocalization Image Creator plugin), and (2) facilitate semi-automatic identification and quantification of colocalized objects (the Colocalization Object Counter plugin). We developed a Microsoft Excel macro script for importing, summarizing, and organizing the OBCA data, and a MATLAB script for visualizing 3D reconstructions of the OBCA data along with tissue contours.

These tools represent a novel platform for OBCA that is notable for its (1) optimization for cell-by-cell analysis, (2) combination of automatic and visually verified object segmentation, thus combining speed and accuracy, (3) ease of use with multiple labels, (4) minimization of Z-projection artifacts and artifacts due to transected objects, and (5) provision of a complete workflow for semi-automatic OBCA, including tools for image processing, object identification and quantification, data organization and object-based 3D reconstruction and visualization.

### Comparison with other colocalization analysis platforms

A major difference between our platform and existing OBCA platforms is the divergence from fully automatic analysis^4,12,22,24–33^. For fully automatic OBCA, objects have to be defined either as points or as fully segmented objects. This has been attempted in various ways, but it has been difficult to develop a universal algorithm that functions across diverse image conditions, and the accuracy rate rarely reaches levels that match visually guided manual approaches^15,16^. The Colocalization Image Creator plugin uses a binarization step to separate objects from image background, but in contrast to fully automatic methods, does not depend on fallible automatic segmentation into individual objects. Instead, identification of individual objects is performed using an effective combination of automatic and visually guided manual detection using the Colocalization Object Counter plugin. This plugin allows users to approach object segmentation and identification either fully manually or fully automatically, or a combination of the two. This broadens the applicability of our tools, in contrast to other tools that are typically designed for more narrowly defined applications. Additionally, since the Colocalization Image Creator plugin enables visualization and element definition to be restricted to cell compartments (such as nuclei), better separation of abutting cells can be achieved, thus improving object separation and identification.

Previously described tools designed for types of use similar to ours, including cell-by-cell analysis and 3D imaging, utilize an automatic object segmentation process that can be adjusted by the user^19,34,35^. However, these rely on segmentation delineations for ultimate quantification of colocalization, which is typically reported by automatically calculated metrics, and are thereby highly sensitive to segmentation accuracy. Moreover, these and other OBCA methods normally require segmentation to be performed on each image channel, which is not always feasible. In contrast, the Colocalization Image Creator can be used with hard-to-binarize images, by allowing up to one image channel to be displayed as a grayscale image/element in each output channel (see examples in Figs. 6, 11, 12C). Overlaying additional grayscale elements in a single output channel is discouraged to avoid colocalization artifacts^12^. However, additional hard-to-binarize image channels can be output separately as demonstrated in Fig. 11B,C from Example 2, which show separate output channels displaying a single grayscale image/element each. Even if none of the image channels can be effectively binarized or segmented (diminishing the benefits of preprocessing with the Colocalization Image Maker), the Colocalization Object Counter, Excel macro, and MATLAB script can still be used to perform OBCA, by manually or semi-automatically identifying objects from the raw image data.

Much effort has focused on the automatic image segmentation problem, particularly as applied to biological tissue^15,16^. This is both a highly valuable and a highly challenging research area. The binarization/segmentation method used in the Colocalization Image Creator is relatively simple, featuring manual or automatic global intensity thresholding. Nevertheless, other image segmentation tools can be integrated easily into the workflow by using them to pre-process images prior to using the Colocalization Image Creator. The ImageJ “Auto local threshold” or “Trainable weka segmentation” plugins are good tools for this.

Supplementary Table S1 online presents a comparison of our platform with several other colocalization platforms. Although not a systematic review of all the available tools for colocalization analysis, the comparison highlights some clear trends in the existing software packages. Almost all packages define objects as fully delineated 2D or 3D objects, only a few of them facilitates manual adjustment of object delineation.

Most packages cannot handle more than two or three input image channels, with the exception of some more advanced packages, such as CellProfiler, TANGO, and QuPath. In these three, colocalization analysis across multiple input channels can be done by measuring pixel values within object delineations across channels, combined with detection threshold levels for defining signal presence. Potential downsides of this approach include a dependency on segmentation accuracy, high signal quality, image resolution, and minimal signal artifacts in all measured channels. These packages are furthermore not optimized for manual inspection or intervention to adjust the output of automated procedures, making the output challenging to verify. These packages can also report distance metrics between objects, which can be used for colocalization analysis between pairs of image channels. However, this requires fully delineated objects in all analyzed image channels, which can be challenging when working with multiple channels.

In summary, the trend for colocalization analysis has been to rely on fully automated segmentation and measurements, and on defining objects by their complete delineation. In contrast, although our method enables an initial definition of objects by full delineation (through binary elements), and includes the option to detect overlapping pixel regions across segmented image channels, this is not mandatory. In the Colocalization Object Counter plugin objects are ultimately defined as single points. Although this eliminates any possibility to perform pixel measurements of complete objects (for which there are many other available packages), it has the advantage of diminishing the importance of segmentation accuracy and signal noise, and makes manual inspection and intervention faster and simpler. Colocalizing objects are defined by the distance between object points, and in practice determined by whether automatically or manually placed points across channels fall within or outside the object's circular overlays of user-defined radius. Moreover, our platform has been designed from the outset with multiple image channels and 3D capabilities in mind.

Many previously described colocalization analysis tools specialize on a specific application within a larger procedure, and sometimes require substantial technical training and experience for installation and efficient operation. We present here an entire workflow, covering all aspects of OBCA, including tools for data organization and for 3D reconstruction of identified objects that requires very little technical training and utilizes widely used and available software. A major benefit of the workflow is that each of the four tools can be used independently, or combined with other tools. In addition to integration with more advanced segmentation methods as mentioned above, the Colocalization Object Counter lends itself well to integration with more advanced automatic detection methods that can be based on ImageJ macros or plugins that output multipoints on image objects. Moreover, the Colocalization Object Counter is designed for use with any image readable by ImageJ, permitting input from other image preprocessing tools or even from raw multi-fluorescence imaging data. Finally, data storage using the common CSV file format allows data files generated by our OBCA pipeline to be input to alternative 3D visualization tools, or conversely our 3D visualization tool can be used with CSV data files generated by other programs.

### Additional features

The Colocalization Image Creator is designed with built-in rules and restrictions to help avoid common errors that can occur during OBCA^3,12^. These include the discouragement of visualizing more than one grayscale element in any given output channel, and particular pixel-mixing rules for binary elements. Nevertheless, the Colocalization Image Creator is developed to be as flexible as possible, allowing users to process multi-label imaging data in a number of different ways, suiting particular experimental conditions or the individual style of the user. Although optimized for use with 3D multicellular imaging data, the plugin is well suited for use with 2D images, and with any type of object, including other cell types, organelles, synapses and even non-biological structures. Furthermore, the Colocalization Image Creator plugin enables saving and loading of employed image processing parameters and plugin settings to a descriptor file, enabling standardized reporting of image processing methods and facilitating reproducibility.

By leveraging a combination of automatic and manual/visual OBCA, accurate, high speed analysis can be achieved. In images of well-separated objects, automatic detection is usually efficient, allowing for hundreds of objects per minute to be quantified. In images where object segmentation is less straightforward, a higher degree of manual verification and adjustment of automatic detection is required, thus decreasing throughput per unit time. However, by becoming proficient with the keyboard shortcuts used to zoom, pan, change image channels, change Z-stack section sequences, manipulate detection markers, and more, high throughput can still be achieved. Moreover, additional time is saved with the efficient data organization tool provided through the Excel macro file, which can quickly summarize large data sets.

### Limitations of the platform

The segmentation of abutting objects, such as touching or overlapping cells, is a particularly difficult problem in the case of certain cell types such as neurons, and is one of the major challenges in OBCA applied to neural tissue. When creating binary elements with our Colocalization Image Creator plugin, abutting cells may present as single objects. This is a limitation in all object segmentation algorithms. However, we encourage the use of three practices to help separate cells accurately. First, if a label is restricted to a cell (or other object) compartment, such as the nucleus, object definition can be restricted to that compartment. When using the nucleus as a marker for neurons, this usually results in greatly improved object separation (compare Fig. 10B,C). Second, the generation of “verification channels” (see “Verification channels” in the “Results” section) can facilitate efficient comparison of binary images/elements with the original image data, allowing for rapid, visually guided manual verification of object definition. Third, depending on object density, Z-projections should be generated from a restricted number of layers along the Z-axis, to avoid excessive object overlap. A Z-stack can be split into smaller sub-stacks in ImageJ using the Image > Stacks > Tools > Make Substack command (see, however, some caveats associated with substacks in the “Results” section entitled “The Z-projection output”).

When using the Colocalization Image Creator to generate processed images, it is important that users are aware that certain settings can lead to the obscurement of objects in the output image. If not taken into consideration, this can significantly confound interpretation. All possible cases of object obscurement stem from the particular pixel-mixing rules for binary elements used (see Figs. 6 and 7). The cases are as follows.

1. When two or more binary elements occupy the same XYZ location in a single output channel, only the element with the highest prioritized color is drawn (see Fig. 6, Supplementary Fig. S2A online). To avoid this problem, (1) limit the number of binary images/elements per channel to one, or (2) limit the number to two binary elements, and enable outlines for one of them. However, if the prioritized binary element represents a “colocalization combination superset” of the deprioritized element, no information is lost following object obscurement. This is demonstrated in example 1 (Fig. 10B), where the white “nucleus AND retrograde AND reporter” labeled objects are prioritized over the green “nucleus AND retrograde” labeled objects.
2. When two or more binary objects occupy the same XY location, but have separate Z locations, only the object closest to the Z-stack top will be drawn in any generated Z-projections (see Fig. 7). Such a selection occurs even in images with a single binary element type (a single color), thus potentially reducing the absolute number of objects drawn in the Z-projection compared to the Z-stack. To better quantify absolute numbers of objects from image Z-stacks, (1) limit analysis to single Z-stack optical slices, or inspect all slices, or (2) depending on object density, generate Z-projections from restricted numbers of optical slices along the Z-axis (substacks). However, it is important to note that if objects are uniformly distributed along the Z-axis and do not vary greatly in size, a selection based on Z-stack position represents an unbiased selection, and preserves the relative ratio of different object types in the Z-projection. If object obscurement becomes excessive, and it is critical to get an accurate absolute count of objects, users should consider stereological approaches^18^.
3. When a binary element is combined with a grayscale element in a single output channel, the binary objects are always prioritized and drawn over any grayscale pixels. To avoid this problem, (1) enable outlines for the binary element, or (2) output the grayscale element to a separate channel.

When using the Colocalization Object Counter plugin, or any semi-automatic OBCA, one drawback is that manual intervention can introduce subjective bias. However, whenever possible, efforts to control for subjectivity should be implemented, such as blinding researchers to the experimental conditions of the image data, or having multiple independent researchers quantify the same data.

Finally, the accuracy of semi-automatic OBCA, both in our and other platforms, crucially depends on the quality and resolution of the raw image data. In cases where image quality is poor, unambiguous object identification may be doomed to fail, whether employing manual or automatic methods. Apart from the ability to pre-process images using ImageJ macro commands, our plugins are not designed to enhance image quality. Best practices for microscopy and image acquisition are therefore always recommended before using our platform or any other colocalization analysis tools^3,12^.

## Conclusion

In conclusion, the tools and workflow we present here will enable researchers to employ OBCA on complex multi-label imaging data in a way that is significantly faster than manual inspection, while maintaining high accuracy that can be difficult to achieve using fully automatic methods. It is our hope that the ImageJ community will be able to build upon and expand the functionalities and advantages of these tools.

## Materials and methods

### ImageJ plugin development

The Colocalization Image Creator and Colocalization Object Counter plugins for ImageJ were implemented in Java Development Kit 8 as ImageJ 1 plugins, ensuring compatibility with all major versions of ImageJ, including Image1, Image2, and FIJI^17,36^. The source code with comments is available at https://github.com/Anders-Lunde/Colocalization_Object_Counter and https://github.com/Anders-Lunde/Colocalization_Image_Creator.

### Excel macro file development

The macro for import, summarization, and export of object quantification data was written in Visual Basic for Application version 7.1 in Microsoft Excel 2010. The source code is embedded in the ColocalizationObjectCounter_Importer.xlsm file, and can be viewed and edited by opening the file in Excel and pressing Alt + F11 to open the code editor.

### MATLAB script development

The MATLAB scripts for visualization of 3D reconstructed data were developed in MATLAB Release 2018b, The MathWorks, Inc., Natick, Massachusetts, United States. The source code embedded in the .m files can be viewed and edited using a text editor or the MATLAB editor, whereas the .fig file that contains the graphical user interface data can be edited with the GUIDE tool in MATLAB.

### Procedures related to generation of sample images

All animal procedures were approved by the Norwegian Animal Research Authority (Forsøksdyrutvalget, FDU ID 8473), were performed in accordance with the regulations of the University of Oslo animal care committee and followed the Federation of European Laboratory Animal Science Associations (FELASA) guidelines. The microscopic images used in examples 1, 2, and 3 were taken from our own research on the molecular determinants of brainstem-to-spinal cord projection neurons^20^. In brief, the samples were generated as follows.

For generating embryonic mice with a red fluorescent protein (tdTomato) expression restricted to a subpopulation of brainstem neurons, the b1r4-Cre transgenic mouse line^37^, which expresses Cre recombinase exclusively in rhombomere 4-derived cells under the control of the Hoxb1 rhombomere 4 enhancer^38^, was crossed with the Ai14 Cre reporter line harboring a loxP-flanked STOP cassette preventing transcription of a CAG promoter-driven tdTomato gene (strain 7914, Jackson Laboratories). Following mating of the two mouse strains, the morning of vaginal plug observation was defined as embryonic day (E)0.5, and embryos were harvested at E16.5. Pregnant dams were anesthetized with isoflurane before cervical dislocation. Dissected embryos were kept in ice-cold (4 °C), oxygenated (95% O_2_–5% CO_2_), artificial CSF [containing the following (in mM): 128 NaCl, 3 KCl, 11 d-glucose, 2.5 CaCl_2_, 1 MgSO_4_, 1.2 NaH_2_PO_4_, 5 HEPES, and 25 NaHCO_3_]. Brain stems with cervical spinal cord attached were dissected out in cold ACSF under a dissection microscope. Mice positive for tdTomato expression were retrogradely labeled from one side of cervical segment 1 with fluorescein-conjugated dextran amine (FDA, 3 kDa; Invitrogen) as described in^20,39^. This results in green fluorescence in a subpopulation of brainstem neurons (bulbospinal projection neurons), which share some overlap with the tdTomato-positive population.

Following retrograde labeling, brain stems were immersion fixed in 4% paraformaldehyde in PBS at 4 °C for 30 min, incubated in 20% sucrose in PBS to equilibration, embedded in Tissue-Tek OCT embedding compound (Sakura), frozen in liquid nitrogen, and sectioned transversely at 20 μm using a cryostat. Note that when analyzing objects smaller than the cellular level, the fidelity of microscopy images may suffer, since fixation for 30 min in 4% paraformaldehyde will not allow fixation sufficient to stop antibody-induced rearrangement of molecules. Sections were processed for immunohistofluorescence as described in^20^. The primary antibody used was guinea pig anti-Lbx1 (gift from Dr. Thomas Müller), which was detected using Cy5 donkey anti-guinea pig IgG (706-175-148, Jacksons, England) as secondary antibody. Staining of nuclei was done with 1 μg/ml Hoechst 33342 (Sigma-Aldrich).

The Z-stack image used in Examples 1 and 2 was acquired with a Zeiss 710 laser confocal microscope (Jena, Germany) using a 40× 1.2 N.A. water immersion objective, and consists of 35 optical slices of 1 μm thickness taken at 0.48 μm intervals, with a XY pixel resolution of 0.21 μm. The Z-stack images used in Example 3 were acquired by tile scanning with a Zeiss 710 laser confocal microscope with a 40× 1.2 N.A. water immersion objective. Each image consists of 28–33 optical slices of 1 μm thickness taken at 0.48 μm intervals, with a XY pixel resolution of 0.21 μm. Excitation and emission ranges used for all images were: Hoechst 405 nm, 419–477 nm; fluorescein 488 nm, 497–545 nm, tdTomato 561 nm, 584–622 nm; Cy5 633 nm, 643–713 nm.

### Fully automated counting with a CellProfiler pipeline

A custom 3D pipeline was created to segment all nuclei into 3D objects, consisting of successively performing RescaleIntensity, Resize (factor = 0.125), MedianFilter (window = 3), Threshold (adaptive, Otsu, default parameters), MedianFilter (window = 3), Watershed (distance, footprint = 10, downsample = 4), MeasureObjectIntensity, ExportToSpreadsheet, ConvertObjectsToImage, SaveImages. A custom ImageJ algorithm was used to extract object volumes. For each of the non-nuclei channels in the Example 3 images (retrograde dextran channel, Hoxb1 channel, Lbx1 channel), the measured raw median signal intensity from within the area of all segmented nuclei, together with object volume was used to determine presence of positive labeling. Minimum median intensity values were 0.04 (retrograde dextran), 0.022 (Hoxb1), and 0.03 (Lbx1), whereas acceptable object volume was between 500 and 12,000 voxels.

## Download and installation

The two ImageJ plugins require downloading and installing ImageJ (https://imagej.nih.gov/ij/download.html), or the recommended and more feature-rich version of ImageJ2 called FIJI (https://imagej.net/Fiji/Downloads). If FIJI is installed, the easiest and best way to install the plugins is to open FIJI and click Help > Update > Manage Update Sites > Add update site, and add https://sites.imagej.net/ObjectColocalizationPlugins/ as the URL. Alternatively, manual installation can be done by downloading the most recent “Colocalization_Object_Counter.jar” and “Colocalization_Image_Creator.jar” files from https://sites.imagej.net/ObjectColocalizationPlugins/plugins/, and saving them to the “/Plugins/” folder of an ImageJ or FIJI installation. After installation, the plugins are available under the “Plugins” menu in ImageJ/FIJI.

The Excel macro file is automatically placed into the “/Counts/” folder where analyzed images are located by the Colocalization Object Counter plugin. Alternatively, the most recent version can be downloaded from https://github.com/Anders-Lunde/Colocalization_Object_Counter. Running the macro requires installation of Microsoft Excel. It has been tested on Microsoft Windows with Excel 2010 and Excel 2016. Earlier versions or other operating systems might not be applicable.

The MATLAB scripts require a MATLAB installation. A full MATLAB license can be purchased from https://se.mathworks.com/products/matlab.html. Alternatively, the latest version of the MATLAB runtime (enables running MATLAB scripts) can be freely downloaded from https://se.mathworks.com/products/compiler/matlab-runtime.html. The MATLAB scripts are automatically placed into the “/Counts/Export/” folder where analyzed images are located by the Colocalization Object Counter plugin. Alternatively, the most recent versions can be downloaded from https://github.com/Anders-Lunde/Colocalization_Object_Counter. Execute the “Colocalization_Visualization_3D_GUI.m” file to start a version with a graphical user interface (GUI), compatible with MATLAB version R2018a (9.4) or later. Execute the “Colocalization_Visualization_3D_non_GUI.m” file to start a version without a graphical user interface (GUI), compatible with MATLAB version R2013b (8.2) or later. In this version, visualization parameters can only be adjusted by editing the file in a text editor, as explained by comments in the file.

## Supplementary information

Supplementary Information.

## Data Availability

Source code for the ImageJ plugins is available online on https://github.com/Anders-Lunde/Colocalization_Object_Counter and https://github.com/Anders-Lunde/Colocalization_Image_Creator. The MATLAB and Excel files with embedded source code is available online on https://github.com/Anders-Lunde/Colocalization_Object_Counter. The input image and the Colocalization Image Creator settings file used for Examples of Usage 1 and 2 can be downloaded from the “Examples of Usage data” folder at https://github.com/Anders-Lunde/ Colocalization_Image_Creator (click the button labeled “Clone or download” and click “Download Zip”). The input images and settings file used in Examples of Usage 3 can be downloaded from https://www.dropbox.com/sh/8ah008sgkfffiwx/AAB1ihprbGq2xxRokqqbLra2a?dl=0. Any other data is available on request.
