# Supplementary material for: A versatile toolbox for semi-automatic cell-by-cell object-based colocalization analysis
Source: Sci Rep. 2020 Nov 4;10:19027. doi: 10.1038/s41598-020-75835-7 (PMC7643144; doi:10.1038/s41598-020-75835-7)
Supplement: Supplementary file 1 — Supplementary Information. [file 41598_2020_75835_MOESM1_ESM.docx]

SUPPLEMENTARY INFORMATION

**Manuscript title**

A versatile toolbox for semi-automatic cell-by-cell object-based colocalization analysis

**Author Names and Affiliations**

Anders Lunde^1^, Joel C. Glover*^1^

^1^Dept. of Molecular Medicine, University of Oslo, 0372 Oslo, Norway

**ImageJ plugin 1: Colocalization Image Creator**

**Details of operation - Main menu buttons**

To add *binary* or *grayscale* elements to the output image, click the “Add element” button in the plugin main menu (Figure 3), which opens the *add image element wizard*. Besides the “Add element” button, the plugin main menu contains the following eight buttons. “Redraw output” applies the current element settings to the current open input image, redraws the output. It also displays the user-defined element settings to the ImageJ log window. “Delete last element” removes the most recently created element and redraws the output. “Delete all elements” deletes all elements and redraws a blank output image. “Save settings” saves the current element settings to a user-defined file location. “Load settings” loads a previously saved element settings file, and applies them to any open input image. “Color priority” enables the user to change the order of color priority for binary elements (see “*Element mixing rules*”). “Z-projection options” presents options for how to display the Z-projection in the output image. “Batch processing” opens a menu enabling batch processing of input images. A counter labeled “Current elements”, located below the buttons, displays how many image elements are currently added to the output image.

**Element type 1: Grayscale elements**

Grayscale elements are created in the *grayscale* part of the *add image element wizard*.

Input channels:

Assigning one (or more) input image channel(s), and selecting the colors for their display (Figure 4A). It is best to display grayscale elements in bright colors (brightest to faintest: white, yellow, cyan, green, magenta, red, blue). The menu and logic for grayscale element combination is the same as the ImageJ built-in “Merge channels” tool, in which input image channels assigned to particular colors are mixed according to standard additive RGB rules. Due to caveats associated with combining multiple grayscale channels for colocalization analysis, a warning will be displayed if attempted.

Output image channel:

A drop down menu labeled “Output image channel” sets the output image channel the element is assigned to (Figure 4A).

Add macro commands:

For more advanced users, ImageJ macro code can be added to filter/process the grayscale element before final output, by clicking the “Add macro commands” button, and then typing in commands. The macro commands can be applied selectively to the output Z-projection and/or the output stack by checking the appropriate boxes.

Preview and finish:

Clicking “Preview” or “Finish” prompts the user to set a brightness level for the selected input image channels. If possible, the brightness should be set so that both weak and strong signals are visualized clearly. Upon clicking “Finish”, the wizard will exit, and the grayscale element is added to the assigned output image channel.

Example:

Figure 4B shows an example of an input image channel being transformed to a grayscale element by applying the settings shown in Figure 4A. Note that the ImageJ macro commands have not been applied, since none of the associated checkboxes have been checked.

**Element type 2: Binary elements**

Binary elements are created in the *binary* part of the *add image element wizard*.

Element color:

Set the color of the element output.

Input channels:

In the menu for binary element creation (Figure 5A), either a manual or an ImageJ built-in automatic binarization method is assigned for each selected input channel, which is used to define the employed intensity threshold levels. If manual is selected, an interactive slider is displayed upon clicking *preview* or *finish*, enabling the user to set an appropriate threshold level. When choosing more than one input channel, only the colocalized signals between channels will be output (boolean AND on the binarized input channels).

Convert to outline:

Use to visualize colocalization between this binary elements and other elements. Any colocalized pixels from subsequently added binary or grayscale elements are displayed *inside* these outlines, simulating “inspection within” the binary objects (Figure 6).

Add macro commands:

Click to add custom macro commands. Commands that convert binary images to grayscale images (for example the “mean” filter) can lead to unexpected results and should be avoided. Some useful ImageJ commands that preserve the image as binary include “minimum/maximum/median” filters, “fill holes”, “open”, “close”, “dilate”, “erode”, “watershed”, “remove outliers”, and “denoise”.

Remove connected components:

Enable to remove connected components smaller than the defined value. If the input image is calibrated, the area size cutoff value can be set in calibrated units.

Advanced options:

Under the “Advanced options” menu, additional input channels can be selected for thresholding, and any signal from the binarized channels will be subtracted from the final output element.

It is also possible to leverage the 3D filtering functions of the “3D Objects Counter” plugin. This includes removing binary 3D objects (defined by connected components) based on size, and/or whether they touch any of the XYZ edges. It requires that the “3D Objects Counter” is installed, which comes bundled with the FIJI version of ImageJ, or can be installed manually.

Output channel, preview and finish:

Assigned output channel, preview and finish are selected in the same way as for grayscale elements.

Example:

Figure 5B shows the steps the plugin goes through when creating the binary element using the example settings shown in Figure 5A.

**The Z-projection output**

In the “Z-projection options” in the plugin main menu (“Overlap priority “), the user can select whether objects closest to the top or the bottom output stack image are prioritized (Supplementary Figure S2B).

The plugin options for Z-projections (Supplementary Figure 8B) are: outputting only the Z-stack (no Z-projection image), outputting only the Z-projection image, or both (both is the default). Choosing the method of generating the Z-projection image (maximum (default), average, minimum, sum, standard deviation, median) is also possible, but this only affects grayscale elements.

**Batch processing**

A batch processing menu is accessible by clicking “Batch processing” in the plugin main menu (Supplementary Figure S3). This is used to apply image element settings to a batch of images. The menu enables setting individual brightness and threshold values for images, either by typing in the values manually (“Manual set values”), or by opening highlighted images and adjusting values interactively (“Adjust values for selected files and channels”). The set of brightness and threshold values can be saved and loaded with “Save value set” and “Load value set”. Clicking “Start batch processing” opens the input images one by one and saves the generated output images to the selected output folder.

**ImageJ plugin 2: Colocalization Object Counter**

**Details of operation**

Supplementary Figure S4 shows the Colocalization Object Counter menu, which contains all the buttons needed for operating the plugin. During operation, this menu is displayed next to the image being analyzed

**Manual counting procedure (Set category, add category, delete cell)**

To start using the plugin, the user has to activate one of three modes, selected near the top of the plugin window: “Add category”, “Set category”, or “Delete cell” (Supplementary Figure S4). Additionally, one or more initial colocalization categories must be selected by activating the category checkboxes near the top of the plugin window (Supplementary Figure S4). By default, “Add category” and category 1 is activated on plugin startup.

All three modes rely on the ImageJ “multipoint” tool for marking of image objects, which is activated automatically when the plugin is loaded, or can be activated by clicking the “multipoint” tool icon in the ImageJ toolbar. Multipoints are created sequentially by left-clicking with the mouse on the image, whereas pressing the alt key while left-clicking deletes selected multipoints. Options for multipoint appearance (size, color) are available by double-clicking the multipoint tool icon in the ImageJ toolbar.

A step-by-step flowchart with example usage of the three modes is shown in Figure 8. Below is a description of how to use the different modes, following the examples in Figure 8. The categories assigned and locations of markers in the examples are chosen arbitrarily for illustration purposes.

*Add category (Figure 8A)*

This mode is used to sequentially assign colocalization categories to objects, for example when marking different categories of objects in successive order. Figure 8A - top row: After activating the “Add category” mode and colocalization category 2, three multipoint markers are created in the image by left-clicking with the mouse. Second row: Clicking the green button labeled “Convert multipoints” transforms the markers into circular overlays that display the colocalization category as numbers in the middle of the overlays. Third row: The multipoint markers are cleared by clicking the “Clear multipoints” button, and category 4 is activated. Multipoints are added to indicate locations of category 4 objects. Markers placed *inside* circular overlays indicate colocalization with previously identified objects, whereas markers placed *outside* overlays indicate newly identified objects. Fourth row: Clicking the “Convert multipoints” button transforms the newly identified objects into circular overlays and displays their category, whereas previous overlays containing markers are updated by adding the currently selected category to overlay category. After this operation, we have 5 total objects: one positive for category 2, two positive for category 4, and two positive for both categories 2 and 4.

*Set category (Figure 8B)*

This mode is used to override (set) the category of previously identified objects, by directly assigning categories to overlays. Multipoints created outside of overlays will create new overlays (same behavior as “Add category”). Figure 8B - top row: This example starts with the image and objects that were identified in the last step of the previous example: 5 objects with various colocalization categories. Second row: By activating the “Set category” mode, the active colocalization category is changed from 4 to 7. Next, two multipoint markers are created inside existing overlays, and one outside. Third row: Clicking “Convert multipoints” replaces the previously assigned categories of the two overlays with category 7, whereas the marker that was outside any overlays is transformed into a new overlay of category 7.

*Delete cell (Figure 8C)*

This mode is used to delete the circular overlays. It functions by placing multipoints inside overlays, which will be deleted upon clicking the “Convert multipoint” button. Alternatively, the “freehand” ImageJ tool can be used instead of multipoints to assign overlay objects for deletion. The freehand tool is activated in the ImageJ toolbar. By drawing a freehand shape that touches one or more overlays, they become marked, and will be deleted upon clicking the “Convert multipoint” button. Overlays can also be deleted by enabling “Toggle overlays selectable” in the plugin menu, selecting individual overlays, and pressing “delete” or “backspace” on the keyboard.

**Automatic object detection**

The Colocalization Object Counter plugin has two built-in tools for automatic object detection; one for single 2D images, and another for 3D images (Z-stacks). The 2D tool utilizes the built-in ImageJ function “find maxima”, and the 3D tool utilizes the “3D Image Suite –3D maxima finder” plugin.

Tips: Choosing “Find maxima” will apply the detection options and display the automatically marked multipoints. After marking, it can be useful to remove any multipoints located inside or outside already existing overlays by clicking “Toggle multipoints inside/outside/all cells”.

***Saving and loading***

Saving and loading of counts to and from files is done by clicking the “Save counts” or “Load counts” buttons in the plugin menu. To ensure that filenames and filepaths are compatible with subsequent import into the Excel macro file (described later), the plugin enforces specific filenames and filepaths for the data-files. This means that the user does not have to specify a filename or path when loading or saving: instead, the correct data file is automatically inferred from the active image filename and filepath, as explained below:

Upon clicking “Save counts”, the plugin automatically creates a folder named “Counts” in the same directory from which the active image was loaded. Inside the “Counts” folder, another folder named “Celldata” is created, where the count data is saved as a csv file. The data file has the same name as the image file, with an additional .csv extension. For example, if counts data for image “experimentABC5.tif” located in folder “C:/Data/Microscopy/” are saved or loaded, the data filepath will be “C:/Data/Microscopy/Counts/Celldata/experimentABC5.tif.csv”

***Keyboard shortcuts***

Upon plugin startup, the user is given the option to enable a set of keyboard shortcuts that can be helpful during image colocalization analysis. Pressing R or F zooms in or out of the image, respectively, focusing on the current mouse location. When zoomed in, pressing the spacebar and dragging with the left mouse button pans the image. Pressing the individual keys of the sequence QWEASDZXC switches between image channels 1-9. Scrolling the mouse wheel changes the Z-stack section position. Alt + left mouse button deletes multipoints. Using these shortcuts can significantly speed up colocalization analysis.

**Serial 3D reconstruction of objects and tissue contours**

Assigning a reference origin XY coordinate and north direction that is consistent across a series of images allows the Excel macro file to import, rotate, and translate all object data points in a common reference frame. This is crucial for accurate 3D reconstruction. A reference origin is an XY-point that can be recognized in all images, for example a point along the midline or along an edge of sectioned tissue. This point will be the coordinate origin (x=0, y=0). A reference north is a line that defines an angle of rotation that can be recognized in all images, for example the midline or a tissue edge. To assign these variables for an image with the Colocalization Object Counter, the user first selects the ImageJ “Line tool” in the tool bar. The user then draws a line, starting at the reference origin, and pointing towards an assigned “up” or “north” direction. After drawing the line, the user clicks “Save origin and north (for 3D reconstruction)” to automatically save the information to a file.

To draw a section or tissue contour, the user first selects the ImageJ “Freehand tool” in the tool bar. The user then draws the contour, and clicks “Save contours (for 3D reconstruction)” to automatically save the information to a file. In the Excel macro file, the XY-coordinates of the contours are also rotated and translated according to the origin and north, to align with the object data. The contour data is used during 3D reconstruction with the Matlab script (described later).

**Excel macro file for data organization**

To import count data into the Excel file (“ColocalizationObjectCounter_Importer.xlsm”), place the file in the “Counts” folder for the respective image series. It will then be able to automatically locate and read any data files it needs. All data files are stored in CSV format.

After opening the Excel file, click “Enable macros” if a security warning is displayed.

The “User input” sheet contains three buttons with associated import/export functions. Cells highlighted in yellow designate values and options that can be adjusted and chosen by the user.

***Button1 (“Check and validate data”)***

Button 1 checks for data files present in the “Counts” folder, displays image filenames found, and whether any 3D data is associated with the images. To assign a Z-axis level to each image, the user can input the Z-distance between images in cell D5 and click button 1. The macro will suggest Z levels for each image based on alphabetized filenames in column G. If any Z levels are incorrect, or total manual input is desired, Z levels can be input directly in column G after clicking button 1.

Note that all X, Y and Z values in the Excel macro are shown as uncalibrated distance values, i.e. in image pixel distances. This means that user-assigned Z-axis distances also have to be indicated in pixel distances for accurate 3D reconstruction. For example, if the Z-distance between two images is known to be 100 μm, this value has to be converted into pixel distance. To do this, it is necessary to know the real dimensions of image pixels (in XY-units). If microscope images come with metadata, the Excel file will display the XY-unit in columns I and J. If not, the value has to be determined manually, by measuring a known distance in the image and dividing by the number of pixels. The XY-unit can then be used to convert real distances to pixel distances. The following formula can be used for this (also shown in the Excel file)

Pixel distance = real distance / XY-unit

Example with 100 μm distance between images and XY unit = 0.313 μm/pixel:

Pixel distance = 100 μm / 0.313 μm/pixel

Pixel distance = 319.5 pixels

Accordingly, 319.5 should be the “Z-distance between images” assigned to cell D5.

***Button 2 (“Import and display data”)***

After assigning Z levels, users can use button 2 to flip images by inverting X/Y coordinates, or to add jitter (randomness) to Z level coordinates to avoid a “sectioned” appearance in the 3D reconstructions. “Invert Y” is set to "yes" by default, since ImageJ starts with the Y-axis value 0 at the top of images. Jitter in Z level coordinates is useful for spreading objects between Z levels that would otherwise be densely clustered in single XY planes, disrupting 3D visualization in side views. Options for adjusting how cell slice information is calculated is available through additional settings.

Clicking button 2 will import object and contour data from saved data files, transform these according to reference origin and north, assign a Z value, and display individual data points in rows in the “Cell data” sheet and “Contour data” sheet. The individual columns show filename, untranslated and translated XY coordinates, Z level with and without jitter, and category information for each counted object. Coordinates of contours are similarly listed in rows, but without any category designation (since contours are not categorized like objects).

***Button 3 (“Export and summarize combined data”)***

Before summarizing and exporting data, the user can use button 3 to edit data points in the “Cell data” and “Contour data” sheets. This enables correction of mistakes without having to re-analyze images with the Colocalization Object Counter plugin. It also allows the user to limit 3D reconstructions to selected sets of data points or images. To remove data points, full rows should be deleted completely, without leaving blank rows intermingled (that is, do NOT use the “clear” function, only the “delete” function). This is because the macro will stop at the first blank row it finds.

After button 3 is pressed, data are read from the “Cell data” and “Contour data” sheets, summarized in the “Data summary” sheet, and exported as files in the “\Counts\Export” folder. The “Data summary” sheet shows total counts of each object category, and for each image file. The “Cell data” is exported as a CSV file named “cell_coordinates.csv”, where commas separate the x, y, z, and category values of each counted object. The “Contour data” is exported similarly as a CSV file named “outline_coordinates.csv”, but without the category values. CSV is a standard file format accepted by many data processing and visualization applications, including Excel and Matlab.

**Matlab script for 3D visualization**

Running the script requires loading of two input CSV files: “cell_coordinates.csv”, which contains the object data, and “outline_coordinates.csv” which contains the tissue contour data. They are both generated by the Excel macro (see previous section) upon clicking button 3, and automatically saved to the “/Counts/Export” folder. It is therefore recommended to copy all the Matlab related files to the “/Counts/Export” folder, and run the “Colocalization_Visualization_3D_GUI.m” file from there. This will enable automatic loading of the input data files without any user input, since they are located in the same folder.

The “colors.txt” file is used to assign unique colors to objects of different categories, and can be edited in a text editor by changing the red/green/blue color values of the categories. The association between colors and object categories is displayed in a text box after clicking the “Visualize” button. Additional options for visualization include setting object size, shape, and outlines. The contour data is visualized as a transparent alphashape, which is a bounding area or volume that envelops a set of 3D points. Changing the alpha value adjusts how loosely or tightly the shape wraps around the contour points. The recommended starting alpha value is approximately half the width (in pixels) of a typical contour outline. Clicking the “Visualize” button will display the 3D figure in a new window. The 3D figure can be rotated, zoomed, panned and more by activating the appropriate buttons in the window. The 3D figure can be inspected from different angles and the views saved as image files using the menu. Advanced users can generate animated movies from the figure by downloading additional scripts or screen recording software online. High quality images can be generated with the Matlab command print('-dpdf','-r1200'), in which the first argument sets the filetype, and the second sets the resolution in dots per inches.

**Tips and hints:**

**Working with large images**

When working with large image files, it is recommended to use a powerful computer with sufficient memory. Make sure you have the latest version of ImageJ and that ImageJ has been assigned enough memory by adjusting Edit -> Options -> Memory & Threads. Generating output and testing different settings with the Colocalization Image Creator from large images can be slow. To speed up the process, the input image can be cropped beforehand. This is recommended when designing new element settings, and when verifying previous settings on new images.

**Notes on reusing, adjusting, and verifying threshold and brightness level settings for batch processing:**

When you reuse element settings, you should verify that these are suitable for your new images. It is useful to perform an optional cropping to speed up processing during adjustment. Ideally, all input images in a series should be processed with the same threshold and brightness levels. Variable brightness settings can influence the automatic detection processes when using the “find maxima” functionality of the Colocalization Object Counter plugin, by affecting the sensitivity of the noise level and pre-blur settings for grayscale elements. Moreover, it is generally better for manual inspection to be consistent in the use of brightness settings, so as not to evaluate different images under different conditions. Variable threshold levels should also be avoided since this effectively changes the definition of objects across images. However, if images have been acquired with different microscope or imaging parameters, or some sections have different levels of background and staining intensity, individual threshold and brightness levels can be set. We emphasize that this should be avoided so as to not compromise analysis reproducibility. Levels can be adjusted for the currently opened input image by clicking the appropriate buttons in the lower part of the main menu. If these levels are to be applied to the uncropped images during batch processing, the levels should be recorded.

**Supplementary Figures**

**Supplementary Figure S1. Colocalization Image Creator plugin main menu.** The plugin main menu consists of a few intuitively labeled buttons. Clicking the “Add Element” button displays a wizard menu with which either a binary or a grayscale element can be generated.


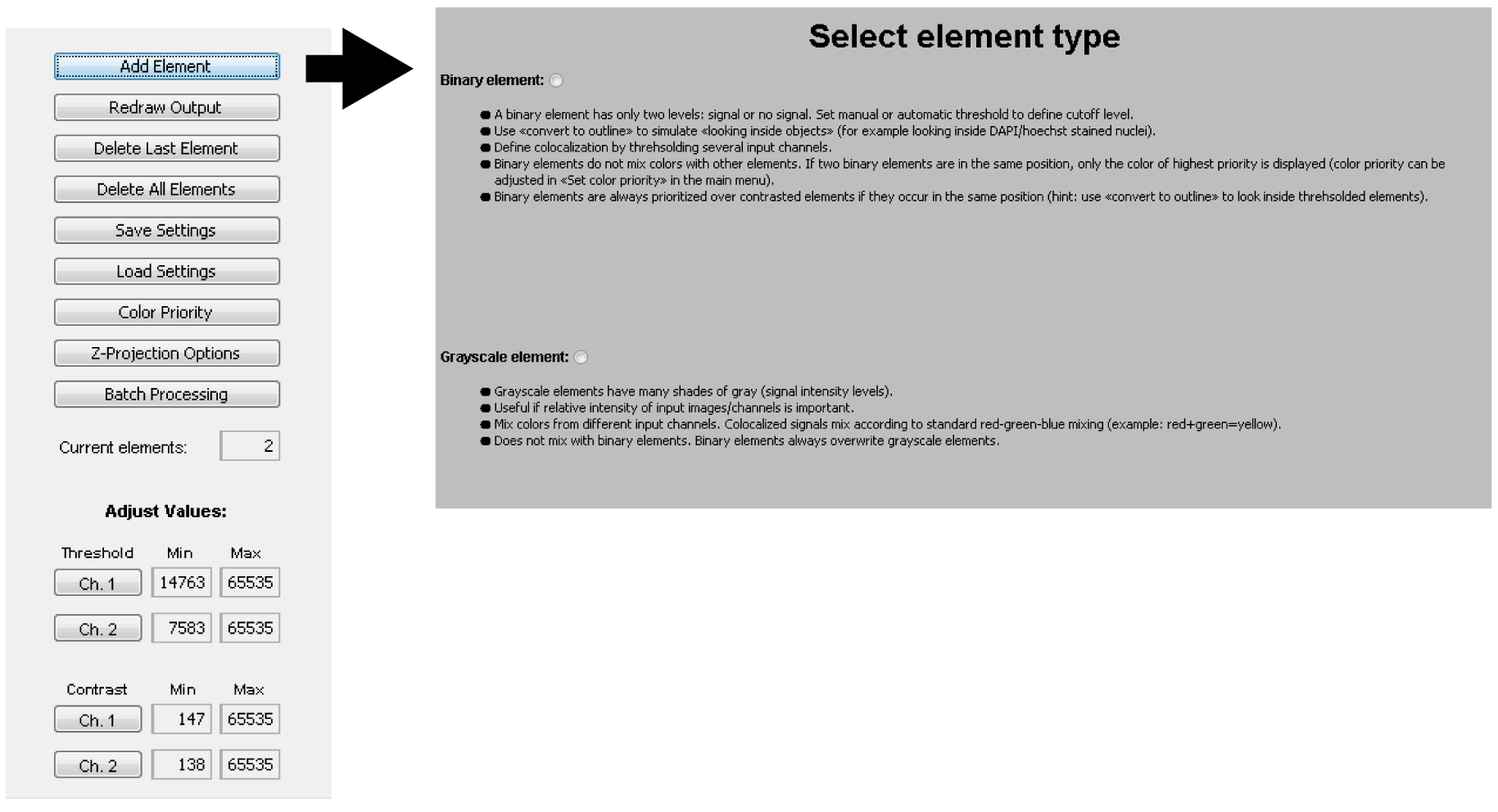


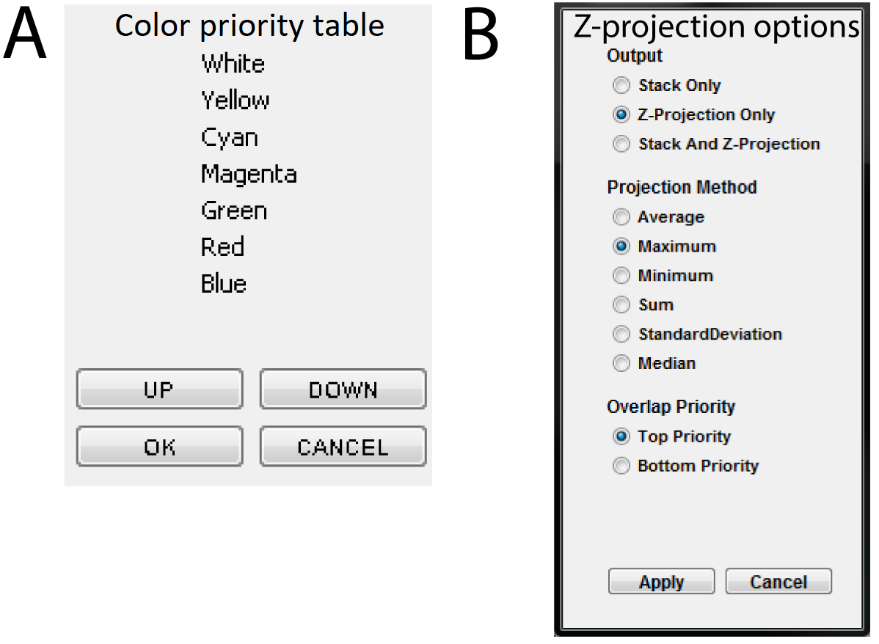


**Supplementary Figure S2. Options and settings for the Colocalization Image Creator plugin.** (A) The color priority table shows the default settings, but can be changed by opening the “Color priority table” option under the plugin main menu. (B) Z-projection options menu available from the plugin main menu. Note that the projection method only affects grayscale elements.


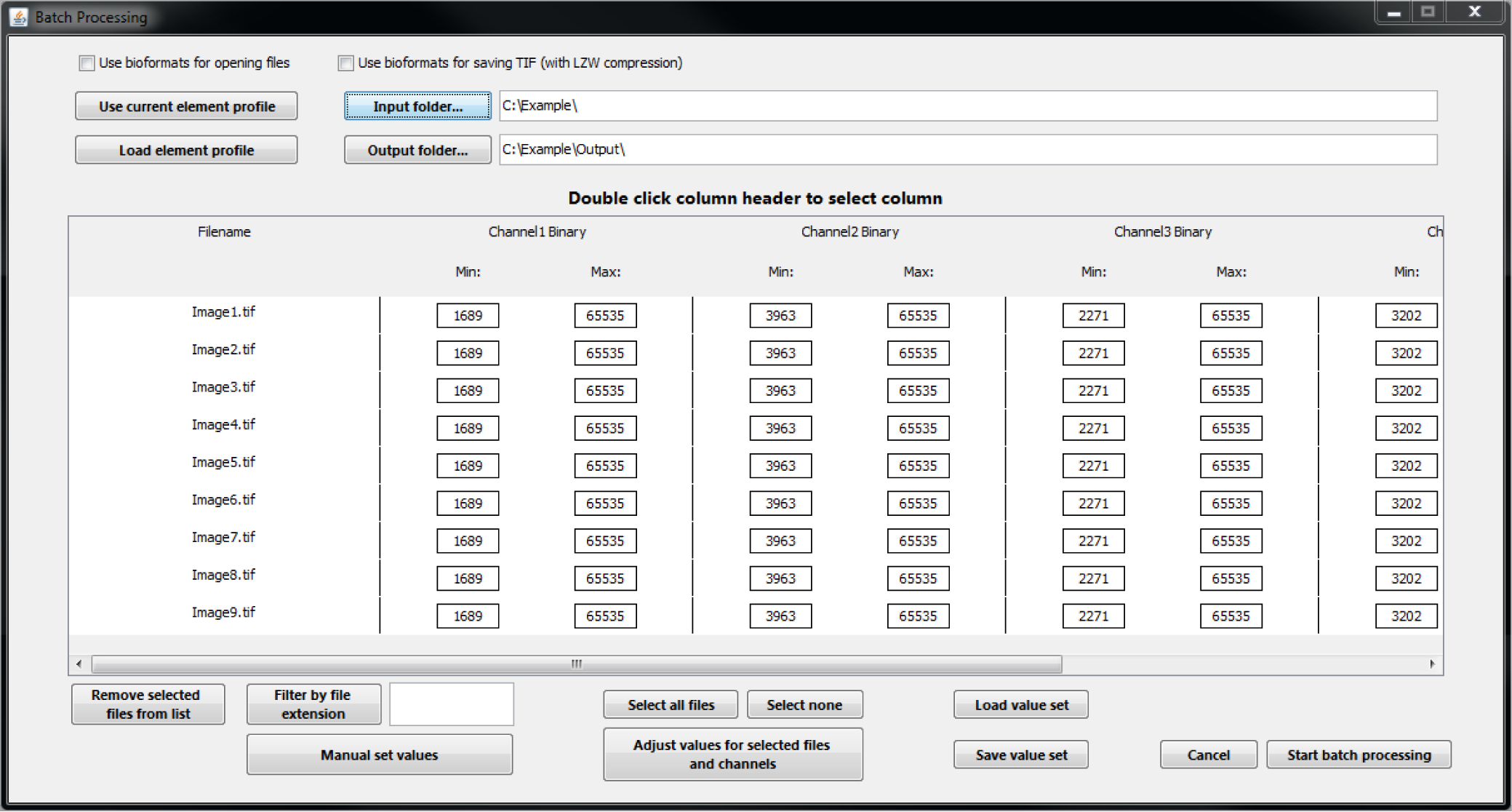


**Supplementary Figure S3. Colocalization Image Creator batch processing menu.** This menu enables the processing of a batch of images with selected element settings. Individual threshold and brightness values can be set for images, and saved and loaded for recordkeeping.


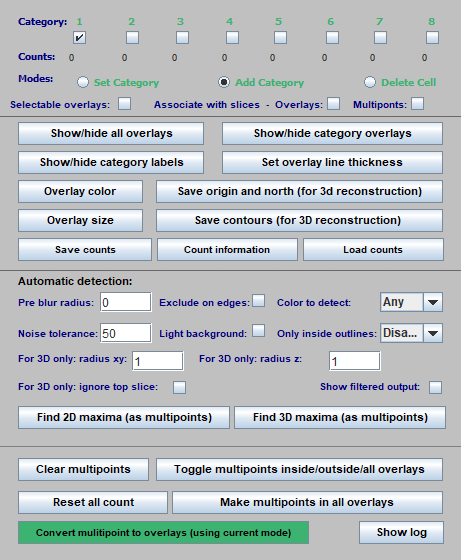


**Supplementary Figure S4. Colocalization Object Counter plugin main menu.** The menu has a simple and intuitive interface, with self-explanatory button labels. Active categories and counting mode are selected with checkboxes near the top of the menu. Options for overlay size and colors, saving/loading counts, and tools for serial 3D reconstruction are in the middle of the menu. Options for automatic object detection are in further down in the menu, and options for converting multipoints to permanent overlays are at the very bottom of the menu.


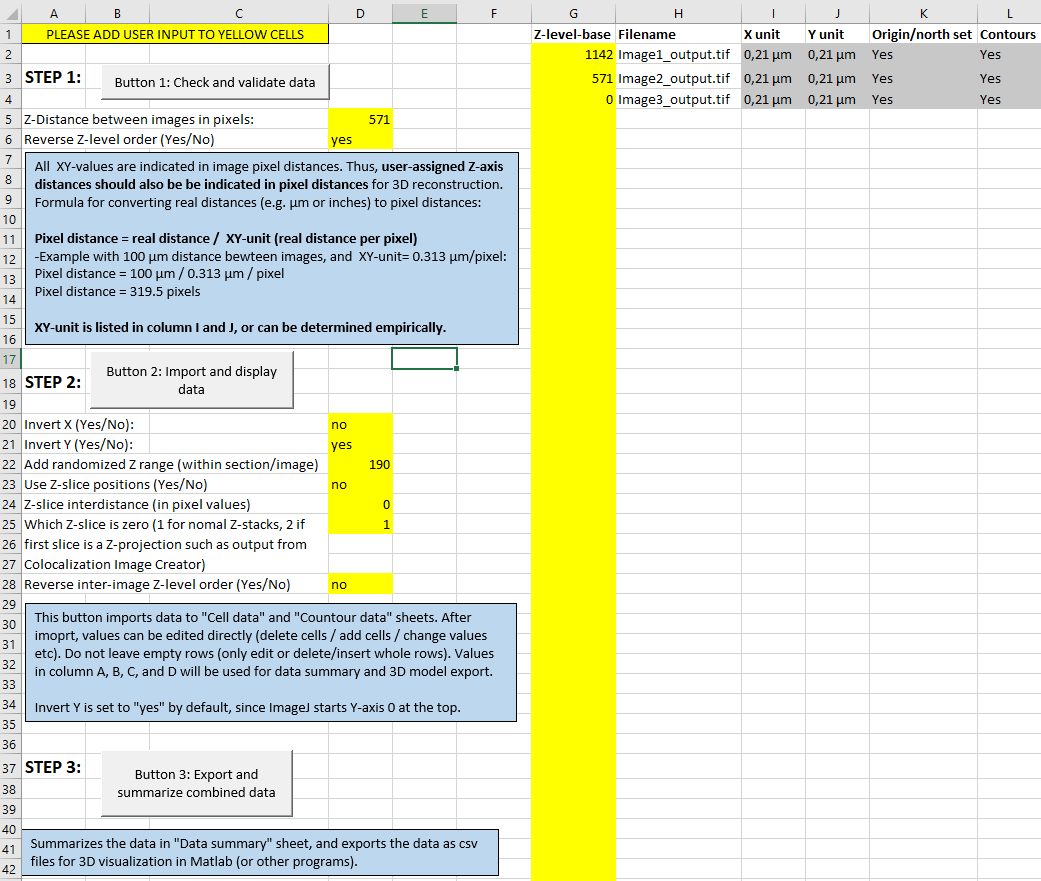


**Supplementary Figure S5. Excel macro file for importing, summarizing, and exporting combined colocalization data.** The Excel macro file automatically reads data files created with the Colocalization Counter plugin, and summarizes the input. It also translates the data to a common origin and reference north, and exports the data for subsequent 3D visualization. The yellow cells represent user input cells. The three buttons are used for validating, importing, and summarizing/exporting the data.


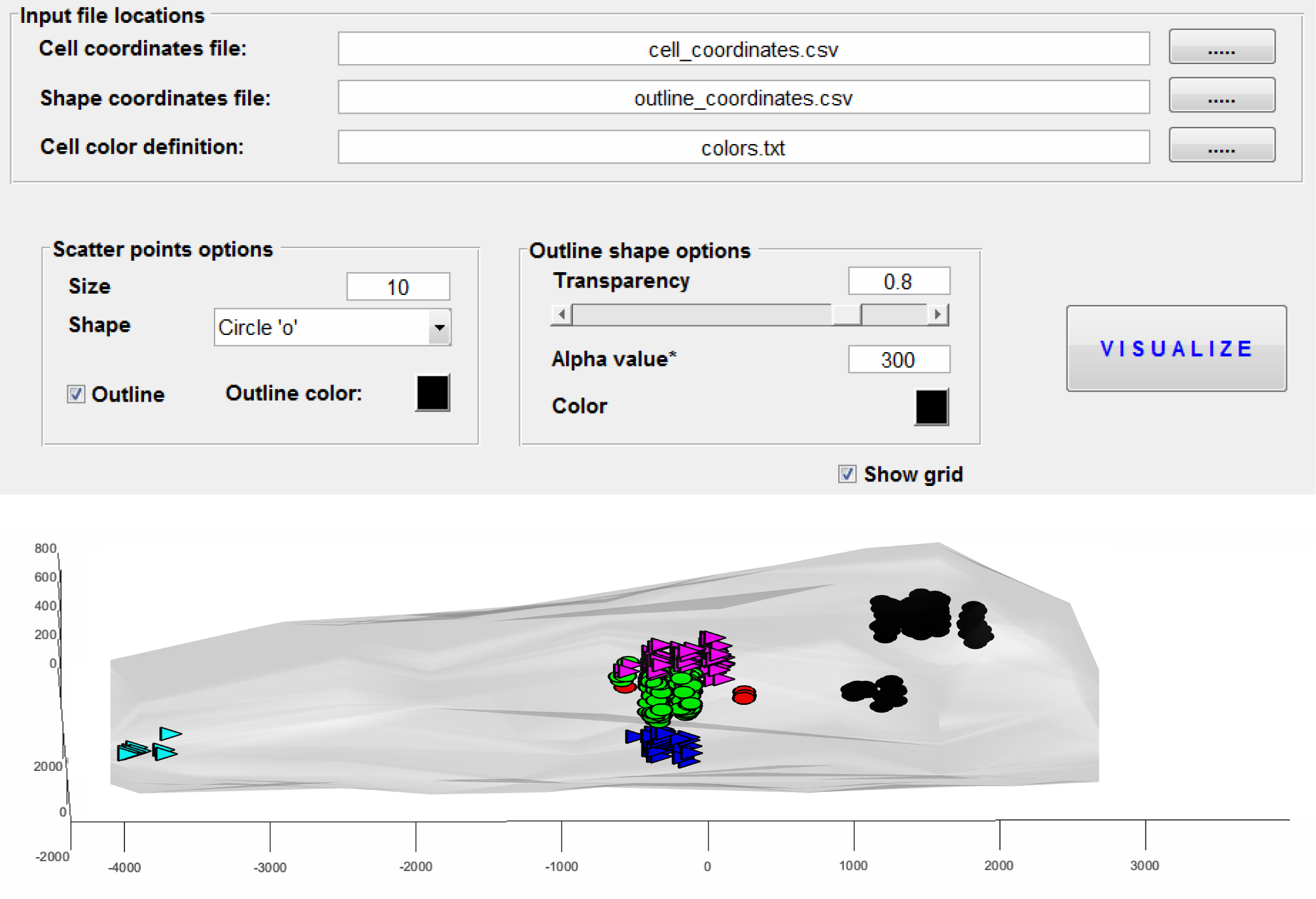


**Supplementary Figure S6. Matlab script graphical menu and example of 3D visualization.** The menu for 3D visualization contains three buttons for loading appropriate input data files. Additional buttons set options for marker color and size, and for transparency and alpha values of the alphashape (tissue outline). Clicking “Visualize” processes the data, and opens an interactive 3D reconstruction, with object categories having distinct colors.

| Software/article name | A versatile toolbox for cell-by-cell object-based colocalization analysis | DiAna | EzColocalization | A Novel Method for Quantified, Superresolved, Three-Dimensional Colocalisation of Isotropic, Fluorescent Particles | 3D Geometry-Based Quantification of Colocalizations in Multichannel 3D Microscopy Images of Human Soft Tissue Tumors | Automated high through-put colocalization analysis of multichannel confocal images |
| --- | --- | --- | --- | --- | --- | --- |
| Reference # | This article | 24 | 25 | 27 | 29 | 31 |
| 3D capabilities | Designed for 3D | Designed for 3D | 2D only | Designed for 3D | Designed for 3D | 2D only |
| Quantification procedure | Semi-automatic | Fully automated | Fully automated | Fully automated | Fully automated | Fully automated |
| Optimized for visual verification | Yes | No | No | No | No | No |
| Object definition | Spots | Fully delineated 3D objects | Fully delineated 2D objects | Spot | Spot with Gaussian sphere. | N/A (Pixel based) |
| Manual intervention | Spot editing | No | no | No | No | No |
| Segmentation method | Global intensity thresholding, Spot segmentation (find maxima) | Global intensity thresholding, Spot segmentation, iterative thresholding | Global intensity thresholding, filtering, watershed. | Custom algorithm. | Custom algorithm. | Global intensity thresholding |
| Colocalization definition | Distance metrics and/or overlap | Distance metrics and/or overlap | Pixel based | Distance metric | Object overlap | Pixel based |
| Multiple fluorescence probes | Multiple | Max 2 | Max 3 | Max 2 | Not reported. At least 3. | Max 2 |
| Platform | ImageJ plugin | ImageJ plugin | ImageJ plugin | Matlab | Not reported | Matlab |

| Software/article name | MatCol: a tool to measure fluorescence signal colocalisation in biological systems | Fluorescence Colocalization Microscopy Analysis Can Be Improved by Combining Object-Recognition With Pixel-Intensity-Correlation | CellProfiler | TANGO: a generic tool for high-throughput 3D image analysis for studying nuclear organization | NEMO: A Tool for Analyzing Gene and Chromosome Territory Distributions From 3D-FISH Experiments | QuPath |
| --- | --- | --- | --- | --- | --- | --- |
| Reference # | 32 | 33 | 22 | 19 | 34 | 35 |
| 3D capabilities | 2D only | 2D only | Some 3D capabilities | Designed for 3D | Designed for 3D | Mainly 2D |
| Quantification procedure | Fully automated | Fully automated | Automatic, or semi-automatic | Fully automated | ? | Automatic, or semi-automatic |
| Optimized for visual verification | No | No | No | No | No | No |
| Object definition | Fully delineated 2D objects | Fully delineated 2D objects | Fully delineated 2D or 3D objects | Fully delineated 3D objects | Fully delineated 3D objects | Fully delineated 2D objects, spots |
| Manual intervention | No | No | Object delineations can be edited | Object delineations can be edited | Manually drawn delineations | Object delineations can be edited, spot editing |
| Segmentation method | Custom algorithm | Global intensity thresholding | Multiple available | Multiple available | Global intensity threshold | Multiple available |
| Colocalization definition | Object overlap | Pixel based | Distance metrics and/or overlap, pixel based metrics | Distance metrics and/or overlap, pixel based metrics | Distance metrics and/or overlap | Distance metrics and/or overlap, pixel based metrics |
| Multiple fluorescence probes | Max 2 | Max 2 | Multiple | Multiple | Max 7 | Multiple |
| Platform | Matlab | ImageJ macro | Custom: CellProfiler | ImageJ and R | Custom: Nemo | Custom: QuPath |

**Supplementary Table S1. Comparison of our OBCA platform to a selection of other relevant platforms.** Of the presented platforms, some distinguishing features of our platform include 3D capabilities, semi-automation, and spot object defenition. Only non-commercial software presented.
